# Automated Machine Learning: A Case Study of Genomic “Image-Based” Prediction in Maize Hybrids

**DOI:** 10.3389/fpls.2022.845524

**Published:** 2022-03-07

**Authors:** Giovanni Galli, Felipe Sabadin, Rafael Massahiro Yassue, Cassia Galves, Humberto Fanelli Carvalho, Jose Crossa, Osval Antonio Montesinos-López, Roberto Fritsche-Neto

**Affiliations:** ^1^Department of Genetics, Luiz de Queiroz College of Agriculture, University of São Paulo, Piracicaba, Brazil; ^2^School of Plant and Environmental Sciences, Virginia Tech, Blacksburg, VA, United States; ^3^Department of Food Engineering, University of Saskatchewan, Saskatoon, SK, Canada; ^4^Centro de Biotecnología y Genómica de Plantas, Universidad Politécnica de Madrid, Madrid, Spain; ^5^International Maize and Wheat Improvement Center (CIMMYT), Texcoco, Mexico; ^6^Facultad de Telemática, Universidad de Colima, Colima, Mexico; ^7^International Rice Research Institute (IRRI), Los Baños, Philippines

**Keywords:** non-image to image, multilayer perceptrons, convolutional neural networks, AutoML, accuracy

## Abstract

Machine learning methods such as multilayer perceptrons (MLP) and Convolutional Neural Networks (CNN) have emerged as promising methods for genomic prediction (GP). In this context, we assess the performance of MLP and CNN on regression and classification tasks in a case study with maize hybrids. The genomic information was provided to the MLP as a relationship matrix and to the CNN as “genomic images.” In the regression task, the machine learning models were compared along with GBLUP. Under the classification task, MLP and CNN were compared. In this case, the traits (plant height and grain yield) were discretized in such a way to create balanced (moderate selection intensity) and unbalanced (extreme selection intensity) datasets for further evaluations. An automatic hyperparameter search for MLP and CNN was performed, and the best models were reported. For both task types, several metrics were calculated under a validation scheme to assess the effect of the prediction method and other variables. Overall, MLP and CNN presented competitive results to GBLUP. Also, we bring new insights on automated machine learning for genomic prediction and its implications to plant breeding.

## Introduction

Genomic prediction (GP) arose as a breeding tool capable of enabling a considerable increase in the rates of genetic gain. In this context, three decades of scientific research have shown that the accuracy of this statistical approach might be conditioned to a series of factors, including the quality and pre-processing of the phenotypic data (Galli et al., 2018), the platform used to obtain genomic information and how it is processed (Granato et al., 2018; Sousa et al., 2019), the population mating design (Fritsche-Neto et al., 2018), the intrinsic genetic architecture of the trait (Alves et al., 2019), the genetic structure of the population (Lyra et al., 2018), how the genotype-by-environment interaction is dealt with (Alves et al., 2021; Costa-Neto et al., 2021), and which prediction methods are used [e.g., BayesA, BayesB (Meuwissen et al., 2001); GBLUP (Bernardo, 1994; VanRaden, 2008); Reproducing Kernel Hilbert Spaces (de Los Campos et al., 2009)].

Several statistical machine learning methods have been adopted for GP because they can help improve genome-enabled prediction accuracy since they are able to make computers learn models or patterns that could be used for analysis, interpretation, prediction, and decision-making. For example, Random Forest (Montesinos-López et al., 2021a), Support Vector Machine (Montesinos-López et al., 2019), and Gradient Boosting Machine (Montesinos-López et al., 2021b). Recently neural networks have been intensively studied and applied in genome-based breeding (Montesinos-López et al., 2019, 2021b). However, one reason why so many types of statistical machine learning methods have been implemented in GP is that no universal best prediction model can be used under all circumstances.

Multilayer perceptrons (MLPs; fully connected layers) and Convolutional Neural Networks (CNNs; fully connected layers and convolutional/pooling filters) are two common types of neural networks (NN). These methods are characterized by the sequentially stacking (several) layers, which automatically identifies latent patterns or features from data (Trevisan et al., 2020). For a technically accurate and contextualized explanation of such models, refer to Pérez-Enciso and Zingaretti (2019). This rising interest is fundamentally associated with the increasing availability of computational power (e.g., graphical processing unit computing, cloud computation, web servers); its success in diverse tasks (such as self-driving vehicles, object detection, and context recognition); ability to work on both regression and classification problems; and especially due to the lower-level restrictions compared to standard models. Also, because neural networks do not need highly pre-processed inputs since these methods are powerful for working directly with raw data (e.g., images, text), and for this reason, they require less human intervention to process data, allowing us to scale machine learning in more interesting ways. For instance, neural networks can perform predictions without restrictive model assumptions; in the context of genetic studies, it does not require specifying the distribution of variables, priors, and the nature of genetic effects (additive, dominance, and epistasis), being theoretically capable of self-adjusting to the underlying genetic architecture (Pérez-Enciso and Zingaretti, 2019).

Initial reports suggest that neural networks can compete with the standard GP methods (e.g., GBLUP) in prediction accuracy. Nevertheless, results are highly inconsistent on this matter (Bellot et al., 2018; Ma et al., 2018; Montesinos-López et al., 2018a,b; Azodi et al., 2019; Abdollahi-Arpanahi et al., 2020), and its best use and performance is still to be determined on a broader and most representative spectrum of prediction scenarios. In this context, one of the major challenges for applying this methodology is identifying adequate model structures and hyperparameters (Bellot et al., 2018; Pérez-Enciso and Zingaretti, 2019; van Dijk et al., 2021). Hereon, we refer to hyperparameter as those not learned with the machine learning algorithm but provided by the user before the learning process of the learnable parameters start, e.g., number of hidden layers, number of neurons per layer, learning rate, filter type, and number, activation function, optimization algorithm, regularization type, etc. Since it is an exceptionally flexible algorithm, there is an infinite number of possible configurations. Therefore, automated procedures are required to explore the possibilities and increase the chance of finding near-to-optimal hyperparameters.

NN’s calibration and training process are very challenging because many hyperparameters need to be selected, and the adequate selection is time-consuming, cumbersome, and complicated. Automated Machine Learning (AutoML) has great potential for identifying adequate network structures and hyperparameters for a given task (Jin et al., 2019). These procedures circumvent hand-designing and testing hyperparameters to save time and effort. Numerous platforms have been developed, such as Auto-sklearn (Feurer et al., 2015), Auto-Weka (Kotthoff et al., 2017), and AutoKeras (Jin et al., 2019); each one with its search algorithm. A comprehensive guide and benchmarking study on the most common search platforms is presented by Truong et al. (2019) for further reference. Despite the importance of hyperparameter tuning and the availability of easy-to-use AutoML tools, the number of reports on its use for identifying artificial neural networks for GP is still very limited (Zingaretti et al., 2020).

Besides adequate hyperparameter tuning, the performance of a neural network is also determined by the quality and preparation of the data fed for training. For example, in neural network-based GP models, the genomic information has been provided as a genomic relationship/distance matrix (Montesinos-López et al., 2018a,b), or as the genomic matrix (Azodi et al., 2019; Abdollahi-Arpanahi et al., 2020). In the case of CNN, the organization of the matrix is meaningful and might contain valuable information (Pérez-Enciso and Zingaretti, 2019). For example, Abdollahi-Arpanahi et al. (2020) applied CNN with genomic matrices to exploit linkage disequilibrium (LD) patterns between genetic markers. In this case, meaningful filter movements were restricted to a single direction (chromosome-wise), seizing physical linkage disequilibrium (e.g., neighboring markers). Nevertheless, LD is known to vary across the genome (Bellot et al., 2018); hence, further advancements to this methodology have been proposed, such as using local convolutional layers applying region-specific filters (Pook et al., 2020).

Recently, a work by Sharma et al. (2019) has shown the possibility of transforming non-image data (e.g., a genomic matrix) into “images” (2 or 3-dimensional visual matrices) leveraging dimensionality reduction techniques. In images, data is coherently distributed along with a space pattern, meaning that neighboring pixels share information in all directions, are correlated (Sharma et al., 2019). The authors reported the superiority of image-based CNN over original data in machine learning tasks and named the pipeline *DeepInsight*. In context, using genomic images would presumably unlock the potential of CNNs for GP, capturing the relationships between markers over new dimensions.

To test new methodologies in a GP context, a key component of comprehensive and meaningful benchmarking starts with an adequate choice of comparison metrics. In this context, regression tasks have mainly relied on metrics such as Pearson’s product–moment correlation and its variations (e.g., divided by the trait’s heritability), Spearman’s correlation, repeatability/heritability, reliability, etc. However, some of these metrics cannot represent the core practice of plant breeding, ranking and selection (Ornella et al., 2014; Blondel et al., 2015). This problem has been tackled using selection-centered metrics, such as selection coincidence (Matias et al., 2017; Galli et al., 2018; Alves et al., 2019). We add to this matter by unifying ranking and selection by discretizing continuous data and conducting prediction based on classification methods suggested by Ornella et al. (2014). This opens the possibility of comparing methods with a new realm of metrics that better align with the context of plant breeding.

AutoML has a full, yet to be determined, potential application for breeding targeted GP. In this context, we present a comprehensive study on using these technologies for predicting plant height (PH) and grain yield (GY) in maize. The objectives of this research were to: (i) assess the comparative performance of MLP and CNN with the standard model GBLUP at predicting PH and GY in maize; (ii) evaluate the performance of neural networks for the GP of PH and GY in maize in regression and classification contexts using MLP and CNN; (iii) elaborate on the use of AutoML to identify the best hyperparameters to perform GP; (iv) and verify the usefulness transforming genomic information into images for CNN-based GP.

## Materials and Methods

### Dependent Variables

#### Field Trials

The genetic material was composed of 904 maize single-cross hybrids obtained from a partial diallel of 49 tropical inbred lines (Fritsche-Neto et al., 2019). Thorough populational description and statistics have been reported on the inbred lines and hybrids (Fritsche-Neto et al., 2018; Alves et al., 2019; Galli et al., 2020a).

The genotypes were arranged in unreplicated trials with the augmented block scheme. Each incomplete block was composed of 18 treatments, 16 regular and two checks (common genotypes). The trials were carried out at Piracicaba-São Paulo (22°42′23″ S, 47°38′14″ W, 535 m) and Anhembi-São Paulo (22°50′51″ S, 48°01′06″ W, 466 m), during the second growing seasons of 2016 (738 hybrids) and 2017 (789 hybrids), under two nitrogen application regimes (ideal: 0.1 Mg ha^−1^ and low: 0.03 Mg ha^−1^). Each experimental unit was composed of a 7 m row. The single-crosses were phenotyped for GY (Mg ha^−1^) and PH (cm). GY was estimated as the production of a plot corrected for 13% moisture. PH was obtained as the mean height, measured from soil to flag leaf, of five plants in the plot.

#### Phenotypic Analysis

The genotypic values of hybrids were obtained with a joint linear mixed model using in ASReml-R (Gilmour et al., 2009) following:


$$y=X\beta +Z_{1}b+Z_{2}g+\varepsilon$$

where y is the phenotype (PH or GY); β is the vector of fixed effects of check, environment (combinations of site, year, and nitrogen regime) and check × environment; $b\sim N\left(0,Ι\sigma_{b}^{2}\right)$ is the random effect of block-within-environment; g $\sim N\left(0,Ι\sigma_{g}^{2}\right)$ is the random effect of regular genotypes (genotypic values); and $\varepsilon \sim N\left(0,diag(\sigma_{1}^{2},\sigma_{2}^{2},\ldots ,\sigma_{8}^{2})\right)$ is a vector of residuals structured by environment estimated from the common treatments (checks). X , $Z_{1}$ , and $Z_{2}$ are the incidence matrices of the mentioned factors. Likelihood Ratio Test (LRT) was used to determine the significance of random effects.

Additionally, a similar model was fit, having check as fixed and regular genotypes, environment, genotype (checks) × environment, and block-within-environment as random for the estimation of variance components. Repeatability at plot level $R_{i}={\hat{\sigma }}_{g}^{2}/\left({\hat{\sigma }}_{g}^{2}+{\hat{\sigma }}_{ga}^{2}+{\hat{\sigma }}_{\varepsilon }^{2}\right)$ was estimated having ${\hat{\sigma }}_{g}^{2}$ , ${\hat{\sigma }}_{ga}^{2}$ , and ${\hat{\sigma }}_{\varepsilon }^{2}$ as the genotypic (hybrids), genotypic (checks) × environment, and residual variances, respectively. The residual variance ( ${\hat{\sigma }}_{\varepsilon }^{2}$ ) was regarded as the mean residual across environments.

#### Genotypic Values Pre-processing

The ultimate goal of plant breeding is ranking and selecting the best genotypes. A common practice is categorizing genotypes in selected and non-selected based on their genetic merit. In this context, the subsequent analysis regards genotypic values as continuous or a discrete variable in two manners. First, genotypic values were categorized based on the absolute values depending on the trait, using two selection intensities (SI), moderate and extreme (Supplementary Figure S1). The moderate SI was created to mimic a balanced dataset regarding the selected and non-selected classes, while the extreme SI was created for an unbalanced dataset. For GY, the higher-yielding individuals were regarded as the best. For the extreme SI, about 10% of the higher-yielding genotypes were selected; and for the moderate SI, around 50% of the higher-yielding were selected for the moderate SI. Second, the selection for PH was based on a hypothetical ideotype. In this case, genotypic values between 1.95 and 2.05 m (~60%; moderate SI) were selected; additionally, genotypic values between 1.90 m and 2.10 (~90%; extreme SI) were regarded as selected. Notice that under the extreme SI, the 10% best were selected for GY, while for PH, the 10% out of type were eliminated. This approach was chosen because selecting the central 10% of hybrids for PH would result in only ~0.02 m range within the “selected” class.

The categorized genotypes were used for classification tasks in the subsequent analysis. For this, the selected genotypes were attributed value 1, while the non-selected had value 0. Notice that the metrics used to evaluate the prediction models might have different meanings for GY and PH. For example, under extreme SI, the individuals regarded as selected compose a low proportion of the samples for GY, while for PH, they are the majority. Finally, the original continuous variables were used in regression tasks. In this case, the genotypic values were scaled using $\hat{g}=\left(\hat{g}-{\hat{g}}_{\min}\right)/\left({\hat{g}}_{\max}-{\hat{g}}_{\min}\right)$ , were ${\hat{g}}_{\min}$ and ${\hat{g}}_{\max}$ are the minimum and maximum genotypic values, respectively.

### Independent Variables

**Figure 1:**
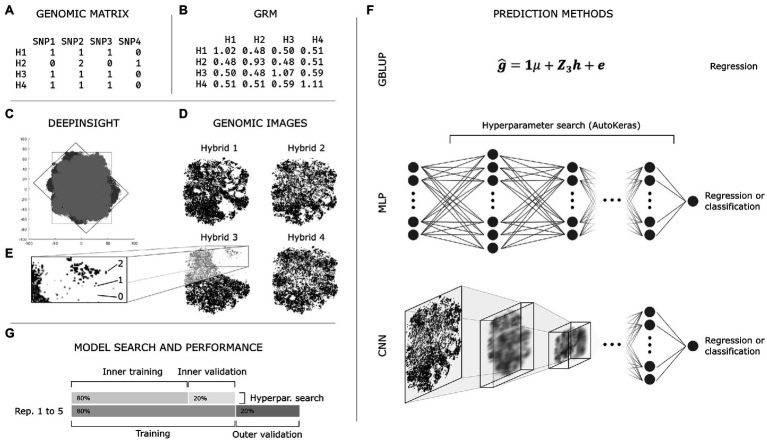
Summarized general exemplification of the employed methodology. **(A)** Genomic data obtained from the pre-processing step; **(B)** Additive Genomic Relationship Matrix (GRM) obtained with VanRaden’s method using the genomic matrix **(A)**; **(C)** *DeepInsight* pipeline: t-SNE decomposition of the genomic matrix **(A)** with original (dark gray) and rotated (light gray) marker coordinates; **(D)** Genomic images obtained with *DeepInsight*; one image is produced for each hybrid and all markers are represented in the image; **(E)** representation of the genotypes (0, 1, and 2, also white, light gray, and dark gray, respectively) in a genomic image; each pixel might comprise a single or multiple markers depending on the level of linkage disequilibrium in the population; **(F)** Genomic prediction methods: genomic BLUP (GBLUP), multilayer perceptron (MLP), and convolutional neural network (CNN); GBLUP and MLP used the GRM **(B)** as independent variable, while CNN used genomic images **(D)**; the neural networks were used as both regression and classification tasks; AutoKeras was used for hyperparameter search in MLP and CNN; **(G)** Simplified representation of the nested validation procedure.

A graphical summary of the procedures explained hereon is presented in Figure 1.

#### Genomic Data Pre-processing

The parental inbred lines were genotyped with the Affymetrix^®^ Axiom^®^ Array of 614 k SNPs (Unterseer et al., 2014). The genomic data pre-processing was performed following the procedure presented in Galli et al. (2020a) by: removing markers with low call rate (<95%); removing markers with at least one heterozygote in the population; imputing missing (homozygous) data with the Synbreed-R package (Wimmer et al., 2012); pruning with Plink v. 1.9 (Chang et al., 2015) so the maximum linkage disequilibrium between markers is 0.9 to avoid high-level redundancy between marker information; building the hybrids synthetic genomic matrix; and, removing markers with minor allele frequency lower than 5%. After pre-processing, a total of 34,571 markers remained for further analysis. Principal component analysis (Lyra et al., 2017; Morosini et al., 2017), linkage disequilibrium decay (Morosini et al., 2017), distribution of minor allele frequency, and heterozygosity (Galli et al., 2020a) have been reported for this dataset.

#### Genomic Relationship Matrix

The genomic information was transformed into two types of data for inclusion in prediction methods. The first type utilized was the additive GRM. We opted for VanRaden's (2008) baseline method to determine the genomic relationship between genotypes. The relationship was obtained as $G=\frac{XX^{\prime }}{trace\left(XX^{\prime }\right)/n}$ , where X is the scaled matrix of genotypic information and n is the number of individuals. The GRM was obtained using the *G.matrix* function of the *snpReady* (Granato et al., 2018) R library.

#### Obtaining Images From Genomic Data

The second type of data transformation was performed by converting the structured genotype matrix by marker into images. This was achieved using the *DeepInsight* algorithm proposed by Sharma et al. (2019). In summary, the algorithm applies a similarity measuring/dimensionality reduction technique (e.g., t-SNE, kPCA) to obtain a Cartesian representation of the similarity between genomic markers in the population. At this step, one graph is produced, and each point represents a marker (Figure 1C, dark gray). In this context, if two markers are somehow related due to, e.g., linkage disequilibrium, they should have similar coordinates. Then, the algorithm finds the smallest rectangle containing all the points and applies a rotation to the graph, so the rectangle is vertically or horizontally oriented (Figure 1C, light gray). At this point, the graph is converted to an image, and the genomic marker information (e.g., 0, 1, or 2, according to the number of copies of the most frequent allele) is mapped to its corresponding position (Figure 1E). This procedure produces one image per hybrid (Figure 1D).

Using *DeepInsight*, images were generated for the 904 genotypes (Figure 1D). The genomic matrix mapped to images had 0, 1, and 2 coding (Figure 1E), commonly used to estimate additive effects of markers or additive GRMs in genomic prediction. The Cartesian plane marker coordinates were estimated using kPCA and t-SNE; no relevant difference was found on preliminary tests, and the latter was selected. The 120 × 120 pixels resolution presented adequate results regarding image size, given the number of markers and the available computational power. The *DeepInsight* algorithm is implemented in MATLAB and available at http://www.alok-ai-lab.com.

### Genomic Prediction

#### Prediction Scenarios

The GP methods used were GBLUP (standard method), MLP (using the GRM), and CNN (using the genomic images obtained with *DeepInsight*; Figure 1F). GP was performed as regression and classification tasks, i.e., the dependent variable (GY or PH) was continuous or discrete, respectively. For the regression task, the evaluated scenarios were: (1) GBLUP; (2) MLP; and (3) CNN. For the classification task, the scenarios were: (1) MLP under moderate SI; (2) MLP under extreme SI; (3) CNN under moderate SI; and (4) CNN under extreme SI. Thus, these scenarios enabled estimating the effect of prediction methods (GBLUP vs. MLP vs. CNN) in the regression task; the impact of selection intensity (moderate vs. extreme) in the classification task; and the effect of and data type/prediction method [MLP (GRM) vs. CNN (genomic image)] on both regression and classification.

#### GBLUP

GBLUP is a standard regression task and was performed using ASReml-R (Gilmour et al., 2009) following the given linear model:


$$\hat{g}=1\mu +Z_{3}h+e$$

where $\hat{g}$ is the scaled vector of genotypic values of hybrids; μ (\mu) is the overall mean; $h\sim N\left(0,G\sigma_{h}^{2}\right)$ is the vector of genomic estimated breeding values, considering that G is the VanRaden's (2008) additive relationship matrix; and e $\sim N\left(0,I\sigma_{e}^{2}\right)$ is the residual vector; 1 vector of one for the intercept and $Z_{3}$ is the incidence matrix for genotypes.

#### Neural Networks

We call the attention that this work is not focused on an in-depth explanation of neural networks as an algorithm despite the need for a basic understanding of neural networks. If the reader is not familiar with the subject, we encourage the reading of González-Camacho et al. (2016) and Pérez-Enciso and Zingaretti (2019) for a thorough comprehension of key concepts.

Neural networks were performed for regression and classification. The python AutoML system *AutoKeras* (Jin et al., 2019) was used in this context. AutoML libraries perform neural architecture search with minor manual intervention and enable the automated finding of population-specific machine learning models. In this context, regression was implemented with the *ImageRegressor* and the *StructuredDataRegressor* functions to search suitable CNNs and MLPs, respectively. The loss function was the mean squared error (MSE; $\;\frac{1}{N}\overset{N}{\underset{i=1}{\sum }}e_{i}^{2}$ ) with $e_{i}$ computed as the difference between observed and predicted values and the metrics were: (a) the mean absolute error (MAE; $\frac{1}{N}\overset{N}{\underset{i=1}{\sum }}\left|e_{i}\right|$ ), and (b) Pearson’s product–moment correlation (r; $\overset{N}{\underset{i=1}{\sum }}\left(gt_{i}-\mu_{gt}\right)\left(gp_{i}-\mu_{gp}\right)/\sqrt{\overset{N}{\underset{i=1}{\sum }}{\left(gt_{i}-\mu_{gt}\right)}^{2}\overset{N}{\underset{i=1}{\sum }}{\left(gp_{i}-\mu_{gp}\right)}^{2}}$ ), given that $e_{i}=gt_{i}-gp_{i}$ is the residual for hybrid i , $gt_{i}$ is the genotypic value of hybrid i , $gp_{i}$ is the predicted value of hybrid i , N is the number of observations, $\mu_{gt}=\frac{1}{N}\overset{N}{\underset{i=1}{\sum }}gt_{i}$ is the mean of genotypic values, and $\mu_{gp}=\frac{1}{N}\overset{N}{\underset{i=1}{\sum }}gp_{i}$ is the mean of predicted values. Also, it is important to point out that these metrics were computed in training (inner-training), validation (inner-validation), and testing sets (outer-validation).

The classification was performed with the *ImageClassifier* and the *StructuredDataClassifier* functions to identify CNNs and MLPs. In this context, positives ( p ) are genotypes that would have been selected based on their genotypic value, and negatives ( n ) are non-selected genotypes. A loss function and several metrics were estimated based on the number of true positives ( tp ), false positives ( fp ), true negatives ( tn ), and false negatives ( fn ). The loss function was the binary cross-entropy, and the metrics were true negative rate (TNR; tn/n ), precision [ tp/(tp+fp) ], recall (or true positive rate; TPR) [ tp/(tp+fn) ], F_1_ score [ 2tp/(2tp+fp+fn) ], accuracy [ (tp+tn)/(tp+tn+fp+fn) ], balanced accuracy [ (TPR+TNR)/2 ], and area under the receiver operating characteristic curve (AUC). As the datasets were imbalanced, especially for the extreme selection intensity, the weights ( w ) of classes (selected and non-selected) were fed to the model given that $w_{i}=1/f_{i}$ , where $f_{i}$ is the frequency of class i .

For both classification and regression, the maximum number of models tried by AutoKeras was 50; the number of epochs was set to 150; the batch size was set to eight; seeds were utilized for reproducibility. Finally, the objective of the search was to identify the hyperparameters that minimized the validation loss.

#### Classification/Regression Performance

A random sampling validation scheme assessed the prediction performance under each validation scenario (Figure 1G). For the neural networks (MLP and CNN), an adaptation of the validation was applied to steps 1 and 2, enabling the identification of the best set of hyperparameters for each replication within a scenario, a process called inner validation or also named model calibration, similar to the utilized by Montesinos-López et al. (2018a,b). The steps exclusively performed for neural networks are represented by lowercase letters.

#### Model Calibration

The overall validation procedure was performed as follows:

1. Allocation of randomly sampled genotypes to training (80%; TS) and validation sets (20%; *VS*):

- Random assignment of samples from the training set into inner training (ITS; 80%) and inner validation sets (IVS; 20%);
- Identification of the best set of hyperparameters with AutoKeras using the inner training and inner validation sets.

2. Fit model using all individuals from the inner sets (ITS and IVS);
3. Prediction of the outer validation set.
4. Estimation of comparison metrics; and
5. Repeat steps 1–4 five times, considering equal set sampling between scenarios.

Comparisons between scenarios were made using the metrics estimated on the validation process. Values are presented as mean and standard deviation across the five replications.

## Results

### Phenotypic Analysis

According to the joint phenotypic analysis, the plot level repeatabilities were 0.23 for GY and 0.59 for PH, revealing the traits as lowly and moderately heritable, respectively. The LRT test (*p* < 0.05) showed the effects of the environment, block within environment, genotype, and genotype (check) by environment to significantly affect both GY and PH. The BLUPs of GY averaged 6.79 Mg ha^−1^ ranging from 4.90 to 8.36 Mg ha^−1^. For PH, the mean was 199.02 cm, with values varying between 170.69 and 217.74 cm (Supplementary Figure S1). More information on these genotypes can be found in Alves et al. (2019), Fritsche-Neto et al. (2019), and Galli et al. (2020a).

### Regression Performance

The regression metrics for the prediction of PH and GY using GBLUP, MLP, or CNN are presented in Table 1. Considering each prediction scenario (e.g., PH with MLP), the metrics (MAE or MSE) were generally consistent across the inner training, inner validation, and outer validation sets. Therefore, scenario comparisons were performed, having the outer validation set as a reference. The values of MAE varied from 0.0635 to 0.0770 for PH and from 0.0797 to 0.0850 for GY. The loss function (MSE) varied from 0.0083 to 0.0109 for PH and from 0.0114 to 0.0128 for GY. The correlations varied from 0.68 to 0.75 for PH and from 0.53 to 0.59 for GY.

**Table 1:** Regression metrics for dependent variables (DV) plant height (PH) and grain yield (GY) using genomic BLUP (GBLUP), Multilayer Perceptrons (MLP), and Convolutional Neural Networks (CNN).

Scenario		MAE						MSE (loss)						r	
DV	Method	Inner training		Inner validation		Outer validation		Inner training		Inner validation		Outer validation		Outer validation	
PH	GBLUP	0.0607	(0.0006)	-	-	0.0635	(0.0017)	0.0076	(0.0002)	-	-	0.0083	(0.0008)	0.75	(0.03)
	MLP	0.0946	(0.0552)	0.0693	(0.0087)	0.0770	(0.0164)	0.0191	(0.0227)	0.0086	(0.0022)	0.0109	(0.0039)	0.70	(0.06)
	CNN	0.0693	(0.0053)	0.0642	(0.0077)	0.0750	(0.0107)	0.0087	(0.0013)	0.0077	(0.0024)	0.0103	(0.0023)	0.68	(0.08)
GY	GBLUP	0.0736	(0.0009)	-	-	0.0836	(0.0038)	0.0098	(0.0003)	-	-	0.0128	(0.0011)	0.56	(0.05)
	MLP	0.0922	(0.0142)	0.0823	(0.0043)	0.0850	(0.0048)	0.0147	(0.004)	0.0114	(0.0017)	0.0125	(0.0018)	0.53	(0.04)
	CNN	0.0776	(0.0117)	0.0778	(0.004)	0.0797	(0.0057)	0.0106	(0.0031)	0.0102	(0.0014)	0.0114	(0.0012)	0.59	(0.01)

Mean absolute error (MAE), mean squared error (MSE; loss function), and Pearson’s product–moment correlation (*r*) are presented for the inner training, inner validation, and outer validation sets. The values are the mean and standard deviations (in parenthesis) across five replications.

Patterns arose from comparisons between the studied scenarios. Contrasting the GBLUP (standard model) with MLP and CNN, GBLUP yielded superior results across all metrics for PH. For GY, a similar pattern was observed when comparing GBLUP with MLP, except for MSE. However, CNN outperformed GBLUP for this trait concerning all metrics. Regarding MLP or CNN, the latter presented better results for both traits considering all estimated metrics, except for r in PH.

### Classification Performance

The classification metrics for the prediction of PH and GY using MLP or CNN are presented in Table 2 and Supplementary Table S1. Under each prediction scenario, the loss increased from inner training to inner validation to outer validation. Regarding the other metrics (with few exceptions), values were (in average across scenarios) greater in inner training, followed by inner validation and outer validation sets. In order to facilitate the evaluation, comparisons between scenarios were performed using the outer validation set as a reference.

**Table 2:** Classification metrics for dependent variables (DV) plant height (PH) and grain yield (GY) using genomic BLUP (GBLUP), Multilayer Perceptrons (MLP), or Convolutional Neural Networks (CNN) under moderate and extreme selection intensities (SI).

Scenario			TNR		Recall (TPR)		Precision		F1 score		Accuracy		Balanced accuracy		AUC		BC (loss)	
DV	Method	SI	Inner training															
PH	MLP	Extreme	0.91	(0.20)	0.95	(0.05)	0.99	(0.02)	0.97	(0.03)	0.95	(0.06)	0.93	(0.11)	0.94	(0.13)	0.0005	(0.0007)
		Moderate	0.55	(0.17)	0.75	(0.13)	0.72	(0.05)	0.73	(0.05)	0.67	(0.05)	0.65	(0.05)	0.69	(0.07)	0.0014	(0.0001)
	CNN	Extreme	0.75	(0.21)	0.72	(0.23)	0.95	(0.04)	0.81	(0.16)	0.73	(0.22)	0.74	(0.22)	0.77	(0.21)	0.0022	(0.0029)
		Moderate	0.61	(0.12)	0.68	(0.08)	0.72	(0.07)	0.70	(0.07)	0.65	(0.09)	0.64	(0.09)	0.68	(0.12)	0.0014	(0.0002)
GY	MLP	Extreme	0.76	(0.26)	0.91	(0.12)	0.46	(0.27)	0.58	(0.28)	0.78	(0.24)	0.84	(0.17)	0.86	(0.18)	0.0009	(0.0007)
		Moderate	0.72	(0.04)	0.74	(0.01)	0.73	(0.03)	0.73	(0.02)	0.73	(0.02)	0.73	(0.02)	0.80	(0.03)	0.0012	(0.0001)
	CNN	Extreme	0.84	(0.13)	0.88	(0.12)	0.51	(0.32)	0.61	(0.28)	0.85	(0.13)	0.86	(0.12)	0.91	(0.10)	0.0007	(0.0006)
		Moderate	0.74	(0.02)	0.76	(0.02)	0.75	(0.01)	0.75	(0.01)	0.75	(0.01)	0.75	(0.01)	0.83	(0.02)	0.0011	(0.0001)
DV	Method	SI	Inner validation															
PH	MLP	Extreme	0.27	(0.18)	0.94	(0.04)	0.92	(0.03)	0.93	(0.02)	0.88	(0.03)	0.61	(0.08)	0.68	(0.16)	0.3454	(0.0951)
		Moderate	0.50	(0.16)	0.76	(0.12)	0.68	(0.06)	0.71	(0.03)	0.64	(0.04)	0.63	(0.04)	0.70	(0.06)	0.6003	(0.0399)
	CNN	Extreme	0.12	(0.16)	0.99	(0.02)	0.91	(0.03)	0.95	(0.02)	0.90	(0.03)	0.55	(0.07)	0.73	(0.14)	0.2863	(0.0640)
		Moderate	0.47	(0.13)	0.80	(0.10)	0.67	(0.06)	0.73	(0.07)	0.66	(0.08)	0.63	(0.08)	0.70	(0.05)	0.6135	(0.0402)
GY	MLP	Extreme	0.94	(0.05)	0.38	(0.23)	0.37	(0.22)	0.37	(0.21)	0.88	(0.04)	0.66	(0.10)	0.86	(0.08)	0.2621	(0.0755)
		Moderate	0.75	(0.06)	0.71	(0.06)	0.74	(0.05)	0.72	(0.04)	0.73	(0.02)	0.73	(0.02)	0.80	(0.02)	0.5427	(0.0159)
	CNN	Extreme	0.97	(0.02)	0.31	(0.20)	0.44	(0.27)	0.35	(0.21)	0.90	(0.02)	0.64	(0.09)	0.84	(0.07)	0.2651	(0.0508)
		Moderate	0.71	(0.04)	0.72	(0.09)	0.70	(0.02)	0.71	(0.05)	0.72	(0.03)	0.72	(0.03)	0.79	(0.02)	0.5486	(0.0188)
DV	Method	SI	Outer validation															
PH	MLP	Extreme	0.26	(0.21)	0.96	(0.03)	0.92	(0.02)	0.93	(0.01)	0.88	(0.01)	0.61	(0.09)	0.66	(0.11)	0.7612	(0.0904)
		Moderate	0.65	(0.18)	0.57	(0.32)	0.71	(0.05)	0.57	(0.28)	0.59	(0.14)	0.61	(0.07)	0.67	(0.07)	1.1120	(0.4637)
	CNN	Extreme	0.32	(0.27)	0.79	(0.35)	0.90	(0.02)	0.79	(0.29)	0.73	(0.28)	0.55	(0.05)	0.63	(0.08)	1.0639	(0.4752)
		Moderate	0.49	(0.15)	0.63	(0.22)	0.65	(0.06)	0.63	(0.16)	0.58	(0.07)	0.56	(0.04)	0.61	(0.02)	1.0838	(0.4774)
GY	MLP	Extreme	0.95	(0.04)	0.40	(0.23)	0.35	(0.22)	0.37	(0.21)	0.90	(0.03)	0.67	(0.10)	0.71	(0.13)	0.6838	(0.3174)
		Moderate	0.66	(0.10)	0.67	(0.05)	0.67	(0.07)	0.67	(0.04)	0.66	(0.04)	0.67	(0.04)	0.72	(0.04)	2.2193	(2.5988)
	CNN	Extreme	0.97	(0.01)	0.17	(0.13)	0.37	(0.19)	0.22	(0.13)	0.90	(0.01)	0.57	(0.06)	0.72	(0.08)	0.7877	(0.4112)
		Moderate	0.72	(0.07)	0.64	(0.04)	0.70	(0.08)	0.67	(0.03)	0.68	(0.02)	0.68	(0.03)	0.74	(0.04)	1.6882	(0.7615)

True negative rate (TNR), recall (or true positive rate; TPR), precision, F1 score, accuracy, balanced accuracy, AUC, and binary cross-entropy (BC; loss function) are presented for the inner training, inner validation, and outer validation sets. The values are the mean and standard deviations (in parenthesis) across five replications.

The observed values of metrics varied depending on the prediction scenario. The TNR varied from 0.26 to 0.65 for PH and 0.66 to 0.97 for GY; the recall ranged from 0.54 to 0.96 for PH and from 0.17 to 0.67 for GY; the precision presented values from 0.65 to 0.92 for PH and from 0.35 to 0.70 for GY; the F1score showed results from 0.57 to 0.93 for PH and from 0.22 to 0.67 for GY; the accuracy varied largely presenting values from 0.52 to 0.88 for PH and 0.66 to 0.90 for GY; the variation of balanced accuracy varied from 0.55 to 0.61 for PH and from 0.57 to 0.68 for GY; at last, the AUC ranged from 0.61 to 0.67 for PH and from 0.71 to 0.74 for GY.

The effect of selection intensity (moderate and extreme) presented tendencies to the estimated metrics. TNR, precision, recall, and F1 score were higher at extreme selection intensity for PH. For GY, the opposite was observed. The accuracy was higher at extreme selection intensity for both traits. The balanced accuracies using GRM were equal for both selection intensities for PH and GY. However, when CNN was used, this metric was lower at extreme selection intensity for both traits.

At last, regarding the AUC, moderate-intensity presented better values for both traits. Regarding the effect of the prediction method, for predicting PH, using MLP showed better values of precision, accuracy, balanced accuracy, and AUC. However, for recall and F1 score, this was only observed at extreme intensity. For GY, using MLP generally presented better results at extreme selection intensity, while CNN was superior at moderate selection intensity. The exceptions were precision, where image-based models were better for both intensities, and recall, which presented the opposite behavior.

### Automated Machine Learning Model Tuning

The neural network structures that minimized the loss function for each replication under each scenario are presented on Supplementary File S1. The classification scenarios were constitutionally composed of an input layer as the first, a dense layer as the second-last summarizing all the neurons of the previous layer, and an activation layer with the sigmoid function to generate the output probabilities. Similarly, the regression scenarios had an input layer as the first and a dense layer as the last to summarize all neurons to one output. Nevertheless, the network structures were generally different, with few exceptions. Among these coincidences, seven out of nine were of the same task type (regression or classification), four were of the same trait (PH or GY), and three were of the same selection intensity (extreme or moderate). However, the number of parameters varied greatly (from 24,533 to 23,589,764), typically higher when images were used.

Dealing with GRM or images requires networks with specific internal layers. The scenarios with MLPs presented a varying number of dense layers (1 to 4); normalization layers (0 to 4; present in about half of the networks); ReLU activation function (positioned after dense layers except the last one); and dropout (0 to 4; present in about 2/3 of networks). The CNNs were composed of 2-dimensional convolutions (1 to 4 in classifications and 2 to 6 in regressions; present in all networks); normalization layers (0 or 1; present in about 2/3 of the networks); 2-dimensional max/global max or average pooling (0 to 3; present in about 2/3 of networks); dropout (0 to 3; present in about 2/3 of networks); image processing filters (resize, random flip, contrast, rotation, translation, and concatenation; 0 to 4; present in about half of the networks; being more common for PH). Also, ResNet50 and Xception networks appeared within 1/3 of the classification networks (more common for PH).

Finally, the preferred optimizer was Adam; Adadelta and SGD also appeared in a limited number of cases. The most common learning rate was 0.001, followed by 0.01, 0.00001, 0.0001, and 0.1. The dropout regularization had values of 0.5 (most common) and 0.25.

## Discussion

### Regression Analysis – The Standard

Benchmark studies suggest the inconsistent performance of neural networks compared to standard GP methods, which depends on a series of factors. We contrasted our findings to reference studies and explored how these factors might have affected the results in this context. Concerning the regression analysis, the GBLUP method outperformed MLP for both traits. One of the factors reported determining the best methodology is how modeling is performed. GP was carried out as a two-stage analysis. Hence, the genotypic value of hybrids across environments was obtained before prediction. Accordingly, the environmental source of variation was absent and could not be captured by the ML methods. For instance, it has been extensively shown that linear models (e.g., GBLUP or BMTME) tend to be outperformed by MLP in a multi-environmental joint analysis if the genotype by environment factor is not modeled for the prediction of PH and GY in maize. This holds under both single (Montesinos-López et al., 2018b) and multi-trait (Montesinos-López et al., 2018a) modeling contexts. Accordingly, MLP was outperformed by GBLUP for both traits in our study, supporting the suggested effect of modeling to the comparative outcome for the studied GP methods.

The use of CNN presented better results than GBLUP and MLP for predicting GY. This contrasts with the findings of Azodi et al. (2019), who suggested that Ridge regression BLUP, a GBLUP-equivalent method, outperforms both MLP and CNN for predicting several traits on numerous crops, including PH and GY in maize inbred lines. In this case, CNN was the poorest performing method for both traits. The inconsistency between the results of these studies regarding the performance of the CNNs for GY could be attributed to the restrictive search space for hyperparameters given the computational requirements for the analysis of an astonishing amount of studied traits and species by Azodi et al. (2019); we tailored ML models to each scenario within each trait, which is known to improve NN performance (Montesinos-López et al., 2018a). Another factor that might have led to this discrepancy was the use of pre-processed genomic information (genomic images) in our CNNs. They opted for using the raw genomic matrix. At last, inbred lines were used in their work, while hybrids were used in ours; studies suggested that CNNs tend to have better performance (than linear methods) when strong nonlinear (e.g., dominance) effects are present (Bellot et al., 2018; Abdollahi-Arpanahi et al., 2020); which is the case of GY in population we studied (Alves et al., 2019).

Regarding the underperformance of NN methods at predicting PH, tangible reasons could be pointed out. It has been hypothesized that the occurrence of extreme allelic frequencies (e.g., only two genotypes are present for a given *locus*) favors linear models by enabling the capture dominance and epistatic variance (Azodi et al., 2019); however, this does not hold for this dataset (Galli et al., 2020a). Also, PH is predominantly governed by additive allelic interactions (Alves et al., 2019), which enables linear models to capture a considerable proportion of the genotypic variance; nevertheless, regardless of the nature of the effects governing the traits under study, ML should always be (at least) as good as linear models given their ability to model linear relationships (Azodi et al., 2019), which was not the case. At last, a cause could be the number of training samples, which might not have been enough for modeling linear and nonlinear interactions between markers by the NN. This is a problem of common occurrence in plant breeding given the usually low number of samples, a large number of markers, and heterogeneity of data (Abdollahi-Arpanahi et al., 2020; Pook et al., 2020).

Overall, CNN presented better results compared to MLP. This advantage might have been due to the processing of the genomic matrix into the additive GRM, in the case of MLP. In this case, only the linear relationship between genotypes was modeled, which might compromise the potential of MLP to identify nonlinear effects. The genomic matrix could be used for further studies at the expense of computational time to overcome this issue.

### Classification Analysis – The Alternative

Given their complex genetic nature, most plant traits present continuous phenotypes. Moreover, traits that were previously discretized by means of measurement ease, such as resistance to biotic stresses, have had their continuous nature better explored by high-throughput phenotyping (Galli et al., 2020b). Therefore, regression tasks are an adequate fit for genetic analysis, including GP. Nevertheless, plant breeding is globally a classification problem in which genotypes are assigned classes (Ornella et al., 2014), usually selected and non-selected. Hence, we elaborate on this problem, unifying ranking and selection by using classifying predictors. These prediction machines were evaluated using metrics that assess the model’s ability to distinguish which genotypes should be selected.

A critical step on classification tasks is the discretization of the continuous variable; when applicable. Discretizing traits has its inherent degree of subjectivity, regarding, e.g., the number of classes and which threshold values are used to classify the data. Accordingly, these choices have been reported to influence the performance of prediction models (Ornella et al., 2014; González-Camacho et al., 2016). Furthermore, a greater level of subjectivity is introduced when genotype classification as true positives or negatives before the prediction is based on the empirical distribution rather than the absolute value of the trait. Predicting genotypes from a related population, classification would not be tied to the percentiles of the distribution on the training population but to the genotypic values and genetic variants under each class and the genetic similarities across populations. In this context, the algorithm might be targeting, e.g., plants with a height between 1.95 and 2.05 m, but not the 10% or 50% best yielding hybrids since the distribution of genotypic values of a new population is likely to differ from the training population.

The classification problem was approached considering two scenarios: one highly imbalanced, where the size of the classes differed substantially (extreme SI), and one nearly balanced, where each class contained about half of the individuals (moderate SI). Both scenarios are plausible and of common occurrence in plant breeding, depending on the program stage. Nevertheless, imbalanced datasets should be evaluated with further cautiousness (Fernández et al., 2011). TNR, precision, recall, F1 score, accuracy, and AUC are examples of metrics sensitive to class imbalance, meaning their results might not be directly interpretable for comparing predictions with differing selection intensities. This is also evidenced by the discrepancy between the accuracy and the balanced accuracy at extreme selection intensity. The selection intensities presented little influence over the balanced accuracy for the same trait and independent variable, except for GY when images were used.

The balanced accuracy is calculated by averaging the proportion of correct predictions in each class, meaning that the label (selected or non-selected) is not relevant. This metric varied from 0.55 to 0.61 for PH and 0.57 to 0.68 for GY. These results are inconsistent with the regression analysis, which showed higher predictability for PH according to all metrics, following the higher heritability of this trait. We postulate that this is associated with the region of the empirical density of genotypic values from which genotypes were regarded as “selected”. For PH, the distinction between the best and the worst individuals was non-directional, which might have difficulted the distinction between which hybrids should or not be selected by the models. For GY, as the selection is directional, this was not an issue. Overall, balanced accuracies were closer to 0.5 (random guess) than to 1 (all correct) for both traits, meaning that further improvements are required. Nevertheless, our results suggest the possibility of non-directional selection, as for PH, which is highly relevant for breeding programs.

Unlike the regression task, where the use of CNN usually presented the best results between machine learning methods, there was considerable inconsistency regarding the superiority of MLP or CNN in the classification task. The comparative performance of the neural network methodologies seemed highly conditioned to trait and selection intensity. Generally, MLP presented the best results for PH, while for GY, the best method heavily depended on the selection intensity. Therefore, it is reasonable to assume that the discretization process of PH and GY impacted the performance of CNN more than that of MLP; but further investigation is warranted. GP prediction regression tasks with machine learning models are already common, but studies comparing methods for predicting discretized variables are still limited.

### Choosing Machine Learning Architectures

The choice of the neural network hyperparameters has been a critical step for NN-based GP by extensive benchmarking (Azodi et al., 2019). Ergo, network search for a given task and dataset has been applied in recent ML-based GP studies (Montesinos-López et al., 2018b; Azodi et al., 2019; Abdollahi-Arpanahi et al., 2020). However, model tuning has been primarily performed using naïve approaches such as random (values sampled from distribution) or grid (discrete values) search, which may limit the number of hyperparameter combinations based on a set of user-defined *a priori* information (Jin et al., 2019). Due to recent advancements in computer science and technology, less restrictive, free, and easy-to-use hyperparameter search algorithms have been made available. Hence, we used Auto-Keras, an AutoML search algorithm with Bayesian optimization to identify (suitable) models. Overall, the algorithm yielded adequate performing neural networks despite the absence of the commonly required human intervention for adjustments.

It has been previously reported that different neural network hyperparameters can be obtained from network search algorithms for a given task (Bellot et al., 2018; Huang et al., 2020). The neural networks selected by the AutoML algorithm presented idiosyncrasies within replications of the same scenario (File S1). The lack of similarity between structures might arise from the ability of AutoML to adapt the network to the dataset (Jin et al., 2019), which changes due to sampling in repeated validation. Huang et al. (2020) suggest this event to be a consequence of insufficient data, but further confirmation is required. Additionally, this may also be associated with the sampling nature of the hyperparameter search system (Jin et al., 2019). Despite the inconsistency between structures, systematic regularities are suggested by the within scenario low standard deviation of the estimated metrics (Tables 1 and 2). Hence, the networks might be capturing similar features, yielding consistent predictions. This has relevant implications for the choice of (deep) neural networks, meaning that distinct but adequate network structures result in similar outcomes.

Further observations can be drawn from the chosen network structures: (i) Although limited, the cases where structures did match (within and between scenarios) suggest that: type of task (regression or classification) is determinant over structure since most matches were of the same type; matches across traits were common, suggesting that similar sources of information might have been captured, which is probably intrinsically associated to the genetic correlation between PH and GY in maize. (ii) Also, when images were used as the input for prediction, augmentation procedures (e.g., resize, flip, rotation) were allocated in the structure of about half of the chosen models despite the spatial structure in the genomic images created by the decomposition performed by *DeepInsight*; further inferences on this matter would require studying the implications of such procedures to the original images, which is not in the scope of this study. (iii) At last, the depth and number of parameters of the networks within scenarios were highly variable for both MLP and CNN, suggesting that simple architectures were as effective as the more complex ones. Simpler models also have the advantage of being generally quicker to train (van Dijk et al., 2021). (iv) Regarding overfitting, which is the tendency of a model to perform well on training but not on unseen data (van Dijk et al., 2021), some differences in performance could be observed between inner training, inner validation, and outer validation sets, but further investigation would be required to determine their extent and consequences. The dropout regularization, temporarily setting a percentage of random neurons to zero (Srivastava et al., 2014), was present on 2/3 of the chosen models, presumably acting on the overfitting issue (Montesinos-López et al., 2018b).

### Further Considerations

Overall, based on the empirical and experimental evidence, neural networks are especially competitive under the presence of strong nonlinear factors and interactions and hidden relationships between pieces of information. Accordingly, it is also dependent on the population type (e.g., lines or hybrids) and the consequent, non-mutually exclusive, genetic architecture of the trait (Bellot et al., 2018; Abdollahi-Arpanahi et al., 2020). The performance of NN is certainly conditioned on the choice of hyperparameters (Bellot et al., 2018; Zingaretti et al., 2020) and neural network type (MLP or CNN). It depends on how the input data is processed before prediction; consequently, special attention should be given to this step since valuable information could be lost. Also, it is presumably dependent on the number of samples and the sample to parameter ratio (Montesinos-López et al., 2018a; Azodi et al., 2019; Pérez-Enciso and Zingaretti, 2019; Abdollahi-Arpanahi et al., 2020). Therefore, it is the scientist’s discretion to test and identify the best performing method for their task. To this day, the only identified consistency regarding GP benchmarking is that no model performs best for all situations.

From experience, inferences on using images for GP could be drawn. In the original work by Sharma et al. (2019), *DeepInsight* was used for transforming RNA-seq, text, and artificial datasets into images. Our work is the first to apply such methodology in a GP context, and it is noteworthy that: (1) the algorithm can create images of different sizes. Image size, which is a hyperparameter, should be adapted to the available dataset and computational power. With the increasing size of the genomic matrix, there is a greater chance that a considerable amount of information would be lost as correlated markers would be tightly grouped, so larger images should be used (Sharma et al., 2019). Additionally, increasing the size of images consequently increases the number of parameters estimated in the neural network, requiring greater computational power. In this work, using 120 by 120 images seemed to be an adequate fit for ~30,000 genomic markers; (2) different dimensionality reduction techniques can be used: t-SNE and kPCA are implemented in the algorithm, but any other of interest can be implemented; further testing should elaborate on this matter; (3) images can have multiple layers: neural networks can model linear and nonlinear relationships between neurons, including other effects and layers of data, such as dominance, epistasis, g × e, transcriptome, and so on.; (4) the cost–benefit in terms of predictive gain and additional work, the use of images as input is arguable. Nevertheless, the methodology’s potential for GP is unprecedented; (5) simulations should provide new valuable and unbiased information.

At last, we discussed two prediction alternatives: regression and classification. Under the regression context, MLP and CNN presented competitive results. Under the classification context, we expected better performances. Nevertheless, we believe that the latter has great potential for plant breeding since it simplifies the pipeline. Neural networks are self-adaptable and aimed at prediction alone. This statement implies that understanding and exposing the events underlying the relationship between phenotypes and genotypes are not of particular interest but could be done if necessary (Azodi et al., 2020). This also implies that limited genetic knowledge of the trait is not a constraint for prediction. Coupled with a simpler processing, direct classification opens new possibilities regarding selecting traits where the ideotype points to intermediate phenotypes, e.g., plant height, ear height, and flowering time (under some circumstances) in maize. Hence, we believe this methodology deserves attention since it could further enhance the GP pipeline in breeding programs.

## Data Availability Statement

The original contributions presented in the study are included in the article/Supplementary Material, further inquiries can be directed to the corresponding author.

## Author Contributions

GG elaborated on the hypothesis, conducted the analyses, and wrote the manuscript. RF-N, HC, RY, FS, JC, OM-L, and CG contributed to interpreting the results and writing. All authors have read and approved the final manuscript.

## Funding

This work was financially supported by Coordenação de Aperfeiçoamento de Pessoal de Nível Superior – Brasil (CAPES) - Finance Code 001 and Conselho Nacional de Desenvolvimento Científico e Tecnológico (CNPq). Fundação de Amparo à Pesquisa do Estado de São Paulo (FAPESP) and the Bill and Melinda Gates Foundation (BMGF): Grant Number INV-003439 BMGF/FCDO for the financial support. Accelerating Genetic Gains in Maize and Wheat for Improved Livelihoods (AG2MW).

## Conflict of Interest

The authors declare that the research was conducted in the absence of any commercial or financial relationships that could be construed as a potential conflict of interest.

## Publisher’s Note

All claims expressed in this article are solely those of the authors and do not necessarily represent those of their affiliated organizations, or those of the publisher, the editors and the reviewers. Any product that may be evaluated in this article, or claim that may be made by its manufacturer, is not guaranteed or endorsed by the publisher.
