# Supplementary material for: Automated Machine Learning: A Case Study of Genomic “Image-Based” Prediction in Maize Hybrids
Source: Front Plant Sci. 2022 Mar 7;13:845524. doi: 10.3389/fpls.2022.845524 (PMC8936805; doi:10.3389/fpls.2022.845524)
Supplement: Supplementary file 1 [file Table_1.docx]

**Supplemental Table S1**. Populational information and classification metrics for (dependent variables; DV) plant height (PH) and grain yield (GY) using Multilayer Perceptron (MLP) or Convolutional Neural Networks (CNN) under moderate and extreme selection intensities (SI). Mean and standard deviation of negatives ($n$), positives ($p$), false negatives ($fn$), false positives ($fp$), true negatives ($tn$), and true positives ($tp$) are shown for the inner training, inner validation, and outer validation population with five-replications

| Scenario | | | n | | p | | fn | | fp | | tn | | tp | |
| --- | --- | --- | --- | --- | --- | --- | --- | --- | --- | --- | --- | --- | --- | --- |
| DV | Method | SI | Inner training | | | | | | | | | | | |
| PH | MLP | Extreme | 60.00 | (2.35) | 524.00 | (2.35) | 23.80 | (28.5) | 5.40 | (11.52) | 54.60 | (12.78) | 500.20 | (28.92) |
|  |  | Moderate | 232.20 | (6.06) | 351.80 | (6.06) | 90.00 | (46.14) | 104.00 | (41.47) | 128.20 | (41.10) | 261.80 | (42.47) |
|  | CNN | Extreme | 60.00 | (2.35) | 524.00 | (2.35) | 145.40 | (118.28) | 14.60 | (12.52) | 45.40 | (13.35) | 378.60 | (117.47) |
|  |  | Moderate | 232.20 | (6.06) | 351.80 | (6.06) | 114.40 | (29.23) | 90.20 | (24.84) | 142.00 | (30.60) | 237.40 | (25.60) |
| GY | MLP | Extreme | 523.20 | (2.28) | 60.80 | (2.28) | 5.60 | (7.70) | 123.00 | (137.34) | 400.20 | (137.73) | 55.20 | (7.76) |
|  |  | Moderate | 291.60 | (3.51) | 292.40 | (3.51) | 76.60 | (4.34) | 81.00 | (11.34) | 210.60 | (9.58) | 215.80 | (5.36) |
|  | CNN | Extreme | 523.20 | (2.28) | 60.80 | (2.28) | 7.00 | (6.96) | 82.80 | (69.04) | 440.40 | (67.83) | 53.80 | (7.95) |
|  |  | Moderate | 291.60 | (3.51) | 292.40 | (3.51) | 69.60 | (5.90) | 75.20 | (4.82) | 216.40 | (5.64) | 222.80 | (4.76) |
|  |  |  | Inner validation | | | | | | | | | | | |
| PH | MLP | Extreme | 13.20 | (3.27) | 125.80 | (3.27) | 7.20 | (4.55) | 9.80 | (4.15) | 3.40 | (2.51) | 118.60 | (4.88) |
|  |  | Moderate | 58.80 | (3.11) | 80.20 | (3.11) | 19.80 | (10.01) | 29.60 | (11.30) | 29.20 | (8.53) | 60.40 | (7.13) |
|  | CNN | Extreme | 13.20 | (3.27) | 125.80 | (3.27) | 1.80 | (3.03) | 11.80 | (4.21) | 1.40 | (2.07) | 124.00 | (4.47) |
|  |  | Moderate | 58.80 | (3.11) | 80.20 | (3.11) | 16.00 | (7.65) | 31.20 | (6.65) | 27.60 | (8.71) | 64.20 | (10.18) |
| GY | MLP | Extreme | 124.60 | (2.30) | 14.40 | (2.30) | 8.80 | (2.86) | 7.20 | (6.18) | 117.40 | (8.17) | 5.60 | (3.36) |
|  |  | Moderate | 71.00 | (4.90) | 68.00 | (4.90) | 19.60 | (4.10) | 17.60 | (4.45) | 53.40 | (6.02) | 48.40 | (6.47) |
|  | CNN | Extreme | 124.60 | (2.30) | 14.40 | (2.30) | 10.20 | (4.55) | 3.60 | (2.88) | 121.00 | (2.45) | 4.20 | (2.95) |
|  |  | Moderate | 71.00 | (4.90) | 68.00 | (4.90) | 18.60 | (4.72) | 20.40 | (1.52) | 50.60 | (6.39) | 49.40 | (9.1) |
|  |  |  | Outer validation | | | | | | | | | | | |
| PH | MLP | Extreme | 19.80 | (2.28) | 161.20 | (2.28) | 7.00 | (4.85) | 14.40 | (3.36) | 5.40 | (4.83) | 154.20 | (6.50) |
|  |  | Moderate | 72.00 | (7.52) | 109.00 | (7.52) | 48.20 | (38.23) | 25.80 | (14.08) | 46.20 | (9.44) | 60.80 | (34.27) |
|  | CNN | Extreme | 19.80 | (2.28) | 161.20 | (2.28) | 34.20 | (57.01) | 13.80 | (6.26) | 6.00 | (4.64) | 127.00 | (55.94) |
|  |  | Moderate | 72.00 | (7.52) | 109.00 | (7.52) | 39.40 | (20.38) | 35.80 | (9.88) | 36.20 | (14.87) | 69.60 | (26.68) |
| GY | MLP | Extreme | 165.20 | (1.92) | 15.80 | (1.92) | 9.60 | (4.34) | 8.80 | (7.26) | 156.40 | (7.30) | 6.20 | (3.63) |
|  |  | Moderate | 89.40 | (7.02) | 91.60 | (7.02) | 30.60 | (5.41) | 30.20 | (9.81) | 59.20 | (8.64) | 61.00 | (5.79) |
|  | CNN | Extreme | 165.20 | (1.92) | 15.80 | (1.92) | 13.20 | (3.27) | 4.40 | (2.30) | 160.80 | (1.30) | 2.60 | (1.67) |
|  |  | Moderate | 89.40 | (7.02) | 91.60 | (7.02) | 32.80 | (6.14) | 25.40 | (8.02) | 64.00 | (1.58) | 58.80 | (2.86) |
