# Supplementary material for: Automated Machine Learning: A Case Study of Genomic “Image-Based” Prediction in Maize Hybrids
Source: Front Plant Sci. 2022 Mar 7;13:845524. doi: 10.3389/fpls.2022.845524 (PMC8936805; doi:10.3389/fpls.2022.845524)
Supplement: Supplementary file 2 [file Data_Sheet_1.docx]

CLASSIFICATION OF GY USING MLP UNDER EXTREME SELECTION INTENSITY

Replication 1

________________________________________________________________

Layer (type) Output Shape Param #

=================================================================

input_1 (InputLayer) [(None, 904)] 0

_________________________________________________________________

multi_category_encoding (Mul (None, 904) 0

_________________________________________________________________

dense (Dense) (None, 32) 28960

_________________________________________________________________

batch_normalization (BatchNo (None, 32) 128

_________________________________________________________________

re_lu (ReLU) (None, 32) 0

_________________________________________________________________

dense_1 (Dense) (None, 1) 33

_________________________________________________________________

classification_head_1 (Activ (None, 1) 0

=================================================================

Total params: 29,121

Trainable params: 29,057

Non-trainable params: 64

_________________________________________________________________

Replication 2

_________________________________________________________________

Layer (type) Output Shape Param #

=================================================================

input_1 (InputLayer) [(None, 904)] 0

_________________________________________________________________

multi_category_encoding (Mul (None, 904) 0

_________________________________________________________________

normalization (Normalization (None, 904) 1809

_________________________________________________________________

dense (Dense) (None, 512) 463360

_________________________________________________________________

batch_normalization (BatchNo (None, 512) 2048

_________________________________________________________________

re_lu (ReLU) (None, 512) 0

_________________________________________________________________

dropout (Dropout) (None, 512) 0

_________________________________________________________________

dense_1 (Dense) (None, 128) 65664

_________________________________________________________________

batch_normalization_1 (Batch (None, 128) 512

_________________________________________________________________

re_lu_1 (ReLU) (None, 128) 0

_________________________________________________________________

dropout_1 (Dropout) (None, 128) 0

_________________________________________________________________

dropout_2 (Dropout) (None, 128) 0

_________________________________________________________________

dense_2 (Dense) (None, 1) 129

_________________________________________________________________

classification_head_1 (Activ (None, 1) 0

=================================================================

Total params: 533,522

Trainable params: 530,433

Non-trainable params: 3,089

_________________________________________________________________

Replication 3

_________________________________________________________________

Layer (type) Output Shape Param #

=================================================================

input_1 (InputLayer) [(None, 904)] 0

_________________________________________________________________

multi_category_encoding (Mul (None, 904) 0

_________________________________________________________________

dense (Dense) (None, 512) 463360

_________________________________________________________________

batch_normalization (BatchNo (None, 512) 2048

_________________________________________________________________

re_lu (ReLU) (None, 512) 0

_________________________________________________________________

dense_1 (Dense) (None, 32) 16416

_________________________________________________________________

batch_normalization_1 (Batch (None, 32) 128

_________________________________________________________________

re_lu_1 (ReLU) (None, 32) 0

_________________________________________________________________

dropout (Dropout) (None, 32) 0

_________________________________________________________________

dense_2 (Dense) (None, 1) 33

_________________________________________________________________

classification_head_1 (Activ (None, 1) 0

=================================================================

Total params: 481,985

Trainable params: 480,897

Non-trainable params: 1,088

_________________________________________________________________

Replication 4

_________________________________________________________________

Layer (type) Output Shape Param #

=================================================================

input_1 (InputLayer) [(None, 904)] 0

_________________________________________________________________

multi_category_encoding (Mul (None, 904) 0

_________________________________________________________________

dense (Dense) (None, 32) 28960

_________________________________________________________________

re_lu (ReLU) (None, 32) 0

_________________________________________________________________

dense_1 (Dense) (None, 256) 8448

_________________________________________________________________

re_lu_1 (ReLU) (None, 256) 0

_________________________________________________________________

dense_2 (Dense) (None, 32) 8224

_________________________________________________________________

re_lu_2 (ReLU) (None, 32) 0

_________________________________________________________________

dense_3 (Dense) (None, 1) 33

_________________________________________________________________

classification_head_1 (Activ (None, 1) 0

=================================================================

Total params: 45,665

Trainable params: 45,665

Non-trainable params: 0

_________________________________________________________________

Replication 5

_________________________________________________________________

Layer (type) Output Shape Param #

=================================================================

input_1 (InputLayer) [(None, 904)] 0

_________________________________________________________________

multi_category_encoding (Mul (None, 904) 0

_________________________________________________________________

dense (Dense) (None, 32) 28960

_________________________________________________________________

re_lu (ReLU) (None, 32) 0

_________________________________________________________________

dropout (Dropout) (None, 32) 0

_________________________________________________________________

dense_1 (Dense) (None, 256) 8448

_________________________________________________________________

re_lu_1 (ReLU) (None, 256) 0

_________________________________________________________________

dropout_1 (Dropout) (None, 256) 0

_________________________________________________________________

dense_2 (Dense) (None, 32) 8224

_________________________________________________________________

re_lu_2 (ReLU) (None, 32) 0

_________________________________________________________________

dropout_2 (Dropout) (None, 32) 0

_________________________________________________________________

dropout_3 (Dropout) (None, 32) 0

_________________________________________________________________

dense_3 (Dense) (None, 1) 33

_________________________________________________________________

classification_head_1 (Activ (None, 1) 0

=================================================================

Total params: 45,665

Trainable params: 45,665

Non-trainable params: 0

_________________________________________________________________

CLASSIFICATION OF GY USING MLP UNDER MODERATE SELECTION INTENSITY

Replication 1

_________________________________________________________________

Layer (type) Output Shape Param #

=================================================================

input_1 (InputLayer) [(None, 904)] 0

_________________________________________________________________

multi_category_encoding (Mul (None, 904) 0

_________________________________________________________________

dense (Dense) (None, 32) 28960

_________________________________________________________________

re_lu (ReLU) (None, 32) 0

_________________________________________________________________

dropout (Dropout) (None, 32) 0

_________________________________________________________________

dense_1 (Dense) (None, 1) 33

_________________________________________________________________

classification_head_1 (Activ (None, 1) 0

=================================================================

Total params: 28,993

Trainable params: 28,993

Non-trainable params: 0

_________________________________________________________________

Replication 2

_________________________________________________________________

Layer (type) Output Shape Param #

=================================================================

input_1 (InputLayer) [(None, 904)] 0

_________________________________________________________________

multi_category_encoding (Mul (None, 904) 0

_________________________________________________________________

dense (Dense) (None, 256) 231680

_________________________________________________________________

re_lu (ReLU) (None, 256) 0

_________________________________________________________________

dense_1 (Dense) (None, 1) 257

_________________________________________________________________

classification_head_1 (Activ (None, 1) 0

=================================================================

Total params: 231,937

Trainable params: 231,937

Non-trainable params: 0

_________________________________________________________________

Replication 3

_________________________________________________________________

Layer (type) Output Shape Param #

=================================================================

input_1 (InputLayer) [(None, 904)] 0

_________________________________________________________________

multi_category_encoding (Mul (None, 904) 0

_________________________________________________________________

dense (Dense) (None, 16) 14480

_________________________________________________________________

re_lu (ReLU) (None, 16) 0

_________________________________________________________________

dropout (Dropout) (None, 16) 0

_________________________________________________________________

dense_1 (Dense) (None, 256) 4352

_________________________________________________________________

re_lu_1 (ReLU) (None, 256) 0

_________________________________________________________________

dropout_1 (Dropout) (None, 256) 0

_________________________________________________________________

dense_2 (Dense) (None, 32) 8224

_________________________________________________________________

re_lu_2 (ReLU) (None, 32) 0

_________________________________________________________________

dropout_2 (Dropout) (None, 32) 0

_________________________________________________________________

dense_3 (Dense) (None, 1) 33

_________________________________________________________________

classification_head_1 (Activ (None, 1) 0

=================================================================

Total params: 27,089

Trainable params: 27,089

Non-trainable params: 0

_________________________________________________________________

Replication 4

_________________________________________________________________

Layer (type) Output Shape Param #

=================================================================

input_1 (InputLayer) [(None, 904)] 0

_________________________________________________________________

multi_category_encoding (Mul (None, 904) 0

_________________________________________________________________

dense (Dense) (None, 32) 28960

_________________________________________________________________

re_lu (ReLU) (None, 32) 0

_________________________________________________________________

dense_1 (Dense) (None, 256) 8448

_________________________________________________________________

re_lu_1 (ReLU) (None, 256) 0

_________________________________________________________________

dense_2 (Dense) (None, 256) 65792

_________________________________________________________________

re_lu_2 (ReLU) (None, 256) 0

_________________________________________________________________

dense_3 (Dense) (None, 1) 257

_________________________________________________________________

classification_head_1 (Activ (None, 1) 0

=================================================================

Total params: 103,457

Trainable params: 103,457

Non-trainable params: 0

_________________________________________________________________

Replication 5

_________________________________________________________________

Layer (type) Output Shape Param #

=================================================================

input_1 (InputLayer) [(None, 904)] 0

_________________________________________________________________

multi_category_encoding (Mul (None, 904) 0

_________________________________________________________________

normalization (Normalization (None, 904) 1809

_________________________________________________________________

dense (Dense) (None, 16) 14480

_________________________________________________________________

batch_normalization (BatchNo (None, 16) 64

_________________________________________________________________

re_lu (ReLU) (None, 16) 0

_________________________________________________________________

dropout (Dropout) (None, 16) 0

_________________________________________________________________

dense_1 (Dense) (None, 256) 4352

_________________________________________________________________

batch_normalization_1 (Batch (None, 256) 1024

_________________________________________________________________

re_lu_1 (ReLU) (None, 256) 0

_________________________________________________________________

dropout_1 (Dropout) (None, 256) 0

_________________________________________________________________

dense_2 (Dense) (None, 64) 16448

_________________________________________________________________

batch_normalization_2 (Batch (None, 64) 256

_________________________________________________________________

re_lu_2 (ReLU) (None, 64) 0

_________________________________________________________________

dropout_2 (Dropout) (None, 64) 0

_________________________________________________________________

dropout_3 (Dropout) (None, 64) 0

_________________________________________________________________

dense_3 (Dense) (None, 1) 65

_________________________________________________________________

classification_head_1 (Activ (None, 1) 0

=================================================================

Total params: 38,498

Trainable params: 36,017

Non-trainable params: 2,481

_________________________________________________________________

CLASSIFICATION OF PH USING MLP UNDER EXTREME SELECTION INTENSITY

Replication 1

_________________________________________________________________

Layer (type) Output Shape Param #

=================================================================

input_1 (InputLayer) [(None, 904)] 0

_________________________________________________________________

multi_category_encoding (Mul (None, 904) 0

_________________________________________________________________

normalization (Normalization (None, 904) 1809

_________________________________________________________________

dense (Dense) (None, 32) 28960

_________________________________________________________________

batch_normalization (BatchNo (None, 32) 128

_________________________________________________________________

re_lu (ReLU) (None, 32) 0

_________________________________________________________________

dense_1 (Dense) (None, 32) 1056

_________________________________________________________________

batch_normalization_1 (Batch (None, 32) 128

_________________________________________________________________

re_lu_1 (ReLU) (None, 32) 0

_________________________________________________________________

dense_2 (Dense) (None, 1) 33

_________________________________________________________________

classification_head_1 (Activ (None, 1) 0

=================================================================

Total params: 32,114

Trainable params: 30,177

Non-trainable params: 1,937

_________________________________________________________________

Replication 2

_________________________________________________________________

Layer (type) Output Shape Param #

=================================================================

input_1 (InputLayer) [(None, 904)] 0

_________________________________________________________________

multi_category_encoding (Mul (None, 904) 0

_________________________________________________________________

normalization (Normalization (None, 904) 1809

_________________________________________________________________

dense (Dense) (None, 512) 463360

_________________________________________________________________

batch_normalization (BatchNo (None, 512) 2048

_________________________________________________________________

re_lu (ReLU) (None, 512) 0

_________________________________________________________________

dropout (Dropout) (None, 512) 0

_________________________________________________________________

dense_1 (Dense) (None, 1) 513

_________________________________________________________________

classification_head_1 (Activ (None, 1) 0

=================================================================

Total params: 467,730

Trainable params: 464,897

Non-trainable params: 2,833

_________________________________________________________________

Replication 3

_________________________________________________________________

Layer (type) Output Shape Param #

=================================================================

input_1 (InputLayer) [(None, 904)] 0

_________________________________________________________________

multi_category_encoding (Mul (None, 904) 0

_________________________________________________________________

normalization (Normalization (None, 904) 1809

_________________________________________________________________

dense (Dense) (None, 32) 28960

_________________________________________________________________

batch_normalization (BatchNo (None, 32) 128

_________________________________________________________________

re_lu (ReLU) (None, 32) 0

_________________________________________________________________

dense_1 (Dense) (None, 32) 1056

_________________________________________________________________

batch_normalization_1 (Batch (None, 32) 128

_________________________________________________________________

re_lu_1 (ReLU) (None, 32) 0

_________________________________________________________________

dense_2 (Dense) (None, 1) 33

_________________________________________________________________

classification_head_1 (Activ (None, 1) 0

=================================================================

Total params: 32,114

Trainable params: 30,177

Non-trainable params: 1,937

_________________________________________________________________

Replication 4

_________________________________________________________________

Layer (type) Output Shape Param #

=================================================================

input_1 (InputLayer) [(None, 904)] 0

_________________________________________________________________

multi_category_encoding (Mul (None, 904) 0

_________________________________________________________________

dense (Dense) (None, 256) 231680

_________________________________________________________________

re_lu (ReLU) (None, 256) 0

_________________________________________________________________

dense_1 (Dense) (None, 128) 32896

_________________________________________________________________

re_lu_1 (ReLU) (None, 128) 0

_________________________________________________________________

dense_2 (Dense) (None, 32) 4128

_________________________________________________________________

re_lu_2 (ReLU) (None, 32) 0

_________________________________________________________________

dense_3 (Dense) (None, 1) 33

_________________________________________________________________

classification_head_1 (Activ (None, 1) 0

=================================================================

Total params: 268,737

Trainable params: 268,737

Non-trainable params: 0

_________________________________________________________________

Replication 5

_________________________________________________________________

Layer (type) Output Shape Param #

=================================================================

input_1 (InputLayer) [(None, 904)] 0

_________________________________________________________________

multi_category_encoding (Mul (None, 904) 0

_________________________________________________________________

dense (Dense) (None, 512) 463360

_________________________________________________________________

batch_normalization (BatchNo (None, 512) 2048

_________________________________________________________________

re_lu (ReLU) (None, 512) 0

_________________________________________________________________

dense_1 (Dense) (None, 1) 513

_________________________________________________________________

classification_head_1 (Activ (None, 1) 0

=================================================================

Total params: 465,921

Trainable params: 464,897

Non-trainable params: 1,024

_________________________________________________________________

CLASSIFICATION OF PH USING MLP UNDER MODERATE SELECTION INTENSITY

Replication 1

_________________________________________________________________

Layer (type) Output Shape Param #

=================================================================

input_1 (InputLayer) [(None, 904)] 0

_________________________________________________________________

multi_category_encoding (Mul (None, 904) 0

_________________________________________________________________

dense (Dense) (None, 1024) 926720

_________________________________________________________________

re_lu (ReLU) (None, 1024) 0

_________________________________________________________________

dropout (Dropout) (None, 1024) 0

_________________________________________________________________

dense_1 (Dense) (None, 32) 32800

_________________________________________________________________

re_lu_1 (ReLU) (None, 32) 0

_________________________________________________________________

dropout_1 (Dropout) (None, 32) 0

_________________________________________________________________

dense_2 (Dense) (None, 32) 1056

_________________________________________________________________

re_lu_2 (ReLU) (None, 32) 0

_________________________________________________________________

dropout_2 (Dropout) (None, 32) 0

_________________________________________________________________

dense_3 (Dense) (None, 1) 33

_________________________________________________________________

classification_head_1 (Activ (None, 1) 0

=================================================================

Total params: 960,609

Trainable params: 960,609

Non-trainable params: 0

_________________________________________________________________

Replication 2

_________________________________________________________________

Layer (type) Output Shape Param #

=================================================================

input_1 (InputLayer) [(None, 904)] 0

_________________________________________________________________

multi_category_encoding (Mul (None, 904) 0

_________________________________________________________________

normalization (Normalization (None, 904) 1809

_________________________________________________________________

dense (Dense) (None, 64) 57920

_________________________________________________________________

re_lu (ReLU) (None, 64) 0

_________________________________________________________________

dropout (Dropout) (None, 64) 0

_________________________________________________________________

dense_1 (Dense) (None, 1) 65

_________________________________________________________________

classification_head_1 (Activ (None, 1) 0

=================================================================

Total params: 59,794

Trainable params: 57,985

Non-trainable params: 1,809

_________________________________________________________________

Replication 3

_________________________________________________________________

Layer (type) Output Shape Param #

=================================================================

input_1 (InputLayer) [(None, 904)] 0

_________________________________________________________________

multi_category_encoding (Mul (None, 904) 0

_________________________________________________________________

normalization (Normalization (None, 904) 1809

_________________________________________________________________

dense (Dense) (None, 64) 57920

_________________________________________________________________

re_lu (ReLU) (None, 64) 0

_________________________________________________________________

dropout (Dropout) (None, 64) 0

_________________________________________________________________

dense_1 (Dense) (None, 16) 1040

_________________________________________________________________

re_lu_1 (ReLU) (None, 16) 0

_________________________________________________________________

dropout_1 (Dropout) (None, 16) 0

_________________________________________________________________

dense_2 (Dense) (None, 1) 17

_________________________________________________________________

classification_head_1 (Activ (None, 1) 0

=================================================================

Total params: 60,786

Trainable params: 58,977

Non-trainable params: 1,809

_________________________________________________________________

Replication 4

_________________________________________________________________

Layer (type) Output Shape Param #

=================================================================

input_1 (InputLayer) [(None, 904)] 0

_________________________________________________________________

multi_category_encoding (Mul (None, 904) 0

_________________________________________________________________

dense (Dense) (None, 32) 28960

_________________________________________________________________

re_lu (ReLU) (None, 32) 0

_________________________________________________________________

dropout (Dropout) (None, 32) 0

_________________________________________________________________

dense_1 (Dense) (None, 1) 33

_________________________________________________________________

classification_head_1 (Activ (None, 1) 0

=================================================================

Total params: 28,993

Trainable params: 28,993

Non-trainable params: 0

_________________________________________________________________

Replication 5

_________________________________________________________________

Layer (type) Output Shape Param #

=================================================================

input_1 (InputLayer) [(None, 904)] 0

_________________________________________________________________

multi_category_encoding (Mul (None, 904) 0

_________________________________________________________________

dense (Dense) (None, 32) 28960

_________________________________________________________________

re_lu (ReLU) (None, 32) 0

_________________________________________________________________

dense_1 (Dense) (None, 32) 1056

_________________________________________________________________

re_lu_1 (ReLU) (None, 32) 0

_________________________________________________________________

dense_2 (Dense) (None, 256) 8448

_________________________________________________________________

re_lu_2 (ReLU) (None, 256) 0

_________________________________________________________________

dropout (Dropout) (None, 256) 0

_________________________________________________________________

dense_3 (Dense) (None, 1) 257

_________________________________________________________________

classification_head_1 (Activ (None, 1) 0

=================================================================

Total params: 38,721

Trainable params: 38,721

Non-trainable params: 0

_________________________________________________________________

CLASSIFICATION OF GY USING CNN UNDER EXTREME SELECTION INTENSITY

Replication 1

_________________________________________________________________

Layer (type) Output Shape Param #

=================================================================

input_1 (InputLayer) [(None, 120, 120, 1)] 0

_________________________________________________________________

normalization (Normalization (None, 120, 120, 1) 3

_________________________________________________________________

separable_conv2d (SeparableC (None, 118, 118, 32) 73

_________________________________________________________________

separable_conv2d_1 (Separabl (None, 116, 116, 64) 2400

_________________________________________________________________

max_pooling2d (MaxPooling2D) (None, 58, 58, 64) 0

_________________________________________________________________

dropout (Dropout) (None, 58, 58, 64) 0

_________________________________________________________________

flatten (Flatten) (None, 215296) 0

_________________________________________________________________

dropout_1 (Dropout) (None, 215296) 0

_________________________________________________________________

dense (Dense) (None, 1) 215297

_________________________________________________________________

classification_head_1 (Activ (None, 1) 0

=================================================================

Total params: 217,773

Trainable params: 217,770

Non-trainable params: 3

_________________________________________________________________

Replication 2

_________________________________________________________________

Layer (type) Output Shape Param #

=================================================================

input_1 (InputLayer) [(None, 120, 120, 1) 0

_________________________________________________________________

random_flip (RandomFlip) (None, 120, 120, 1) 0

_________________________________________________________________

concatenate (Concatenate) (None, 120, 120, 3) 0

_________________________________________________________________

xception (Functional) (None, 4, 4, 2048) 20861480

_________________________________________________________________

global_average_pooling2d (Globa (None, 2048) 0

_________________________________________________________________

dense (Dense) (None, 1) 2049

_________________________________________________________________

classification_head_1 (Activati (None, 1) 0

=================================================================

Total params: 20,863,529

Trainable params: 20,809,001

Non-trainable params: 54,528

_________________________________________________________________

Replication 3

_________________________________________________________________

Layer (type) Output Shape Param #

=================================================================

input_1 (InputLayer) [(None, 120, 120, 1)] 0

_________________________________________________________________

normalization (Normalization (None, 120, 120, 1) 3

_________________________________________________________________

conv2d (Conv2D) (None, 118, 118, 32) 320

_________________________________________________________________

conv2d_1 (Conv2D) (None, 116, 116, 64) 18496

_________________________________________________________________

max_pooling2d (MaxPooling2D) (None, 58, 58, 64) 0

_________________________________________________________________

dropout (Dropout) (None, 58, 58, 64) 0

_________________________________________________________________

flatten (Flatten) (None, 215296) 0

_________________________________________________________________

dropout_1 (Dropout) (None, 215296) 0

_________________________________________________________________

dense (Dense) (None, 1) 215297

_________________________________________________________________

classification_head_1 (Activ (None, 1) 0

=================================================================

Total params: 234,116

Trainable params: 234,113

Non-trainable params: 3

_________________________________________________________________

Replication 4

_________________________________________________________________

Layer (type) Output Shape Param #

=================================================================

input_1 (InputLayer) [(None, 120, 120, 1) 0

_________________________________________________________________

random_flip (RandomFlip) (None, 120, 120, 1) 0

_________________________________________________________________

random_contrast (RandomContrast (None, 120, 120, 1) 0

_________________________________________________________________

resizing (Resizing) (None, 224, 224, 1) 0

_________________________________________________________________

concatenate (Concatenate) (None, 224, 224, 3) 0 _________________________________________________________________

xception (Functional) (None, None, None, 2 20861480

_________________________________________________________________

global_max_pooling2d (GlobalMax (None, 2048) 0

_________________________________________________________________

dense (Dense) (None, 1) 2049

_________________________________________________________________

classification_head_1 (Activati (None, 1) 0 dense[0][0]

=================================================================

Total params: 20,863,529

Trainable params: 20,809,001

Non-trainable params: 54,528

_________________________________________________________________

Replication 5

_________________________________________________________________

Layer (type) Output Shape Param #

=================================================================

input_1 (InputLayer) [(None, 120, 120, 1) 0

_________________________________________________________________

normalization (Normalization) (None, 120, 120, 1) 3

_________________________________________________________________

random_translation (RandomTrans (None, 120, 120, 1) 0 normalization[0][0]

_________________________________________________________________

concatenate (Concatenate) (None, 120, 120, 3) 0

_________________________________________________________________

resnet50 (Functional) (None, 4, 4, 2048) 23587712

_________________________________________________________________

global_average_pooling2d (Globa (None, 2048) 0

_________________________________________________________________

dense (Dense) (None, 1) 2049

_________________________________________________________________

classification_head_1 (Activati (None, 1) 0

=================================================================

Total params: 23,589,764

Trainable params: 23,536,641

Non-trainable params: 53,123

_________________________________________________________________

CLASSIFICATION OF GY USING CNN UNDER MODERATE SELECTION INTENSITY

Replication 1

_________________________________________________________________

Layer (type) Output Shape Param #

=================================================================

input_1 (InputLayer) [(None, 120, 120, 1)] 0

_________________________________________________________________

normalization (Normalization (None, 120, 120, 1) 3

_________________________________________________________________

conv2d (Conv2D) (None, 118, 118, 32) 320

_________________________________________________________________

conv2d_1 (Conv2D) (None, 116, 116, 64) 18496

_________________________________________________________________

max_pooling2d (MaxPooling2D) (None, 58, 58, 64) 0

_________________________________________________________________

dropout (Dropout) (None, 58, 58, 64) 0

_________________________________________________________________

flatten (Flatten) (None, 215296) 0

_________________________________________________________________

dropout_1 (Dropout) (None, 215296) 0

_________________________________________________________________

dense (Dense) (None, 1) 215297

_________________________________________________________________

classification_head_1 (Activ (None, 1) 0

=================================================================

Total params: 234,116

Trainable params: 234,113

Non-trainable params: 3

_________________________________________________________________

Replication 2

_________________________________________________________________

Layer (type) Output Shape Param #

=================================================================

input_1 (InputLayer) [(None, 120, 120, 1)] 0

_________________________________________________________________

normalization (Normalization (None, 120, 120, 1) 3

_________________________________________________________________

random_flip (RandomFlip) (None, 120, 120, 1) 0

_________________________________________________________________

separable_conv2d (SeparableC (None, 118, 118, 32) 73

_________________________________________________________________

separable_conv2d_1 (Separabl (None, 116, 116, 64) 2400

_________________________________________________________________

flatten (Flatten) (None, 861184) 0

_________________________________________________________________

dropout (Dropout) (None, 861184) 0

_________________________________________________________________

dense (Dense) (None, 1) 861185

_________________________________________________________________

classification_head_1 (Activ (None, 1) 0

=================================================================

Total params: 863,661

Trainable params: 863,658

Non-trainable params: 3

_________________________________________________________________

Replication 3

_________________________________________________________________

Layer (type) Output Shape Param #

=================================================================

input_1 (InputLayer) [(None, 120, 120, 1)] 0

_________________________________________________________________

normalization (Normalization (None, 120, 120, 1) 3

_________________________________________________________________

conv2d (Conv2D) (None, 118, 118, 32) 320

_________________________________________________________________

max_pooling2d (MaxPooling2D) (None, 59, 59, 32) 0

_________________________________________________________________

dropout (Dropout) (None, 59, 59, 32) 0

_________________________________________________________________

flatten (Flatten) (None, 111392) 0

_________________________________________________________________

dropout_1 (Dropout) (None, 111392) 0

_________________________________________________________________

dense (Dense) (None, 1) 111393

_________________________________________________________________

classification_head_1 (Activ (None, 1) 0

=================================================================

Total params: 111,716

Trainable params: 111,713

Non-trainable params: 3

_________________________________________________________________

Replication 4

_________________________________________________________________

Layer (type) Output Shape Param #

=================================================================

input_1 (InputLayer) [(None, 120, 120, 1)] 0

_________________________________________________________________

normalization (Normalization (None, 120, 120, 1) 3

_________________________________________________________________

conv2d (Conv2D) (None, 114, 114, 32) 1600

_________________________________________________________________

conv2d_1 (Conv2D) (None, 108, 108, 128) 200832

_________________________________________________________________

max_pooling2d (MaxPooling2D) (None, 18, 18, 128) 0

_________________________________________________________________

dropout (Dropout) (None, 18, 18, 128) 0

_________________________________________________________________

flatten (Flatten) (None, 41472) 0

_________________________________________________________________

dropout_1 (Dropout) (None, 41472) 0

_________________________________________________________________

dense (Dense) (None, 1) 41473

_________________________________________________________________

classification_head_1 (Activ (None, 1) 0

=================================================================

Total params: 243,908

Trainable params: 243,905

Non-trainable params: 3

_________________________________________________________________

Replication 5

_________________________________________________________________

Layer (type) Output Shape Param #

=================================================================

input_1 (InputLayer) [(None, 120, 120, 1)] 0

_________________________________________________________________

normalization (Normalization (None, 120, 120, 1) 3

_________________________________________________________________

conv2d (Conv2D) (None, 114, 114, 32) 1600

_________________________________________________________________

conv2d_1 (Conv2D) (None, 108, 108, 64) 100416

_________________________________________________________________

max_pooling2d (MaxPooling2D) (None, 18, 18, 64) 0

_________________________________________________________________

flatten (Flatten) (None, 20736) 0

_________________________________________________________________

dropout (Dropout) (None, 20736) 0

_________________________________________________________________

dense (Dense) (None, 1) 20737

_________________________________________________________________

classification_head_1 (Activ (None, 1) 0

=================================================================

Total params: 122,756

Trainable params: 122,753

Non-trainable params: 3

_________________________________________________________________

CLASSIFICATION OF PH USING CNN UNDER EXTREME SELECTION INTENSITY

Replication 1

_________________________________________________________________

Layer (type) Output Shape Param #

=================================================================

input_1 (InputLayer) [(None, 120, 120, 1)] 0

_________________________________________________________________

normalization (Normalization (None, 120, 120, 1) 3

_________________________________________________________________

conv2d (Conv2D) (None, 118, 118, 32) 320

_________________________________________________________________

conv2d_1 (Conv2D) (None, 116, 116, 64) 18496

_________________________________________________________________

max_pooling2d (MaxPooling2D) (None, 58, 58, 64) 0

_________________________________________________________________

dropout (Dropout) (None, 58, 58, 64) 0

_________________________________________________________________

flatten (Flatten) (None, 215296) 0

_________________________________________________________________

dropout_1 (Dropout) (None, 215296) 0

_________________________________________________________________

dense (Dense) (None, 1) 215297

_________________________________________________________________

classification_head_1 (Activ (None, 1) 0

=================================================================

Total params: 234,116

Trainable params: 234,113

Non-trainable params: 3

_________________________________________________________________

Replication 2

_________________________________________________________________

Layer (type) Output Shape Param #

=================================================================

input_1 (InputLayer) [(None, 120, 120, 1) 0

_________________________________________________________________

normalization (Normalization) (None, 120, 120, 1) 3

_________________________________________________________________

random_flip (RandomFlip) (None, 120, 120, 1) 0

_________________________________________________________________

concatenate (Concatenate) (None, 120, 120, 3) 0

_________________________________________________________________

resnet50 (Functional) (None, 4, 4, 2048) 23587712

_________________________________________________________________

global_average_pooling2d (Globa (None, 2048) 0

_________________________________________________________________

dense (Dense) (None, 1) 2049

_________________________________________________________________

classification_head_1 (Activati (None, 1) 0

=================================================================

Total params: 23,589,764

Trainable params: 23,536,641

Non-trainable params: 53,123

_________________________________________________________________

Replication 3

_________________________________________________________________

Layer (type) Output Shape Param #

=================================================================

input_1 (InputLayer) [(None, 120, 120, 1) 0

_________________________________________________________________

normalization (Normalization) (None, 120, 120, 1) 3

_________________________________________________________________

resizing (Resizing) (None, 224, 224, 1) 0

_________________________________________________________________

concatenate (Concatenate) (None, 224, 224, 3) 0

_________________________________________________________________

resnet50 (Functional) (None, None, None, 2 23587712

_________________________________________________________________

global_average_pooling2d (Globa (None, 2048) 0

_________________________________________________________________

dropout (Dropout) (None, 2048) 0

_________________________________________________________________

dense (Dense) (None, 1) 2049

_________________________________________________________________

classification_head_1 (Activati (None, 1) 0

=================================================================

Total params: 23,589,764

Trainable params: 23,536,641

Non-trainable params: 53,123

_________________________________________________________________

Replication 4

_________________________________________________________________

Layer (type) Output Shape Param #

=================================================================

input_1 (InputLayer) [(None, 120, 120, 1) 0

_________________________________________________________________

normalization (Normalization) (None, 120, 120, 1) 3

_________________________________________________________________

random_translation (RandomTrans (None, 120, 120, 1) 0

_________________________________________________________________

random_flip (RandomFlip) (None, 120, 120, 1) 0

_________________________________________________________________

concatenate (Concatenate) (None, 120, 120, 3) 0

_________________________________________________________________

resnet50 (Functional) (None, 4, 4, 2048) 23587712

_________________________________________________________________

global_max_pooling2d (GlobalMax (None, 2048) 0

_________________________________________________________________

dense (Dense) (None, 1) 2049

_________________________________________________________________

classification_head_1 (Activati (None, 1) 0

=================================================================

Total params: 23,589,764

Trainable params: 23,536,641

Non-trainable params: 53,123

_________________________________________________________________

Replication 5

_________________________________________________________________

Layer (type) Output Shape Param #

=================================================================

input_1 (InputLayer) [(None, 120, 120, 1) 0

_________________________________________________________________

normalization (Normalization) (None, 120, 120, 1) 3

_________________________________________________________________

random_translation (RandomTrans (None, 120, 120, 1) 0

_________________________________________________________________

concatenate (Concatenate) (None, 120, 120, 3) 0

_________________________________________________________________

xception (Functional) (None, 4, 4, 2048) 20861480

_________________________________________________________________

flatten (Flatten) (None, 32768) 0

_________________________________________________________________

dropout (Dropout) (None, 32768) 0

_________________________________________________________________

dense (Dense) (None, 1) 32769

_________________________________________________________________

classification_head_1 (Activati (None, 1) 0

=================================================================

Total params: 20,894,252

Trainable params: 20,839,721

Non-trainable params: 54,531

_________________________________________________________________

CLASSIFICATION OF PH USING CNN UNDER MODERATE SELECTION INTENSITY

Replication 1

_________________________________________________________________

Layer (type) Output Shape Param #

=================================================================

input_1 (InputLayer) [(None, 120, 120, 1) 0

_________________________________________________________________

normalization (Normalization) (None, 120, 120, 1) 3

_________________________________________________________________

random_translation (RandomTrans (None, 120, 120, 1) 0

_________________________________________________________________

random_flip (RandomFlip) (None, 120, 120, 1) 0

_________________________________________________________________

concatenate (Concatenate) (None, 120, 120, 3) 0

_________________________________________________________________

resnet50 (Functional) (None, 4, 4, 2048) 23587712

_________________________________________________________________

global_average_pooling2d (Globa (None, 2048) 0

_________________________________________________________________

dense (Dense) (None, 1) 2049

_________________________________________________________________

classification_head_1 (Activati (None, 1) 0

=================================================================

Total params: 23,589,764

Trainable params: 23,536,641

Non-trainable params: 53,123

_________________________________________________________________

Replication 2

_________________________________________________________________

Layer (type) Output Shape Param #

=================================================================

input_1 (InputLayer) [(None, 120, 120, 1)] 0

_________________________________________________________________

normalization (Normalization (None, 120, 120, 1) 3

_________________________________________________________________

separable_conv2d (SeparableC (None, 114, 114, 32) 113

_________________________________________________________________

separable_conv2d_1 (Separabl (None, 108, 108, 64) 3680

_________________________________________________________________

max_pooling2d (MaxPooling2D) (None, 18, 18, 64) 0

_________________________________________________________________

dropout (Dropout) (None, 18, 18, 64) 0

_________________________________________________________________

flatten (Flatten) (None, 20736) 0

_________________________________________________________________

dropout_1 (Dropout) (None, 20736) 0

_________________________________________________________________

dense (Dense) (None, 1) 20737

_________________________________________________________________

classification_head_1 (Activ (None, 1) 0

=================================================================

Total params: 24,533

Trainable params: 24,530

Non-trainable params: 3

_________________________________________________________________

Replication 3

_________________________________________________________________

Layer (type) Output Shape Param #

=================================================================

input_1 (InputLayer) [(None, 120, 120, 1)] 0

_________________________________________________________________

normalization (Normalization (None, 120, 120, 1) 3

_________________________________________________________________

random_translation (RandomTr (None, 120, 120, 1) 0

_________________________________________________________________

conv2d (Conv2D) (None, 118, 118, 32) 320

_________________________________________________________________

max_pooling2d (MaxPooling2D) (None, 59, 59, 32) 0

_________________________________________________________________

flatten (Flatten) (None, 111392) 0

_________________________________________________________________

dropout (Dropout) (None, 111392) 0

_________________________________________________________________

dense (Dense) (None, 1) 111393

_________________________________________________________________

classification_head_1 (Activ (None, 1) 0

=================================================================

Total params: 111,716

Trainable params: 111,713

Non-trainable params: 3

_________________________________________________________________

Replication 4

_________________________________________________________________

Layer (type) Output Shape Param #

=================================================================

input_1 (InputLayer) [(None, 120, 120, 1)] 0

_________________________________________________________________

normalization (Normalization (None, 120, 120, 1) 3

_________________________________________________________________

random_flip (RandomFlip) (None, 120, 120, 1) 0

_________________________________________________________________

random_contrast (RandomContr (None, 120, 120, 1) 0

_________________________________________________________________

conv2d (Conv2D) (None, 118, 118, 16) 160

_________________________________________________________________

conv2d_1 (Conv2D) (None, 116, 116, 64) 9280

_________________________________________________________________

max_pooling2d (MaxPooling2D) (None, 58, 58, 64) 0

_________________________________________________________________

dropout (Dropout) (None, 58, 58, 64) 0

_________________________________________________________________

flatten (Flatten) (None, 215296) 0

_________________________________________________________________

dense (Dense) (None, 1) 215297

_________________________________________________________________

classification_head_1 (Activ (None, 1) 0

=================================================================

Total params: 224,740

Trainable params: 224,737

Non-trainable params: 3

_________________________________________________________________

Replication 5

_________________________________________________________________

Layer (type) Output Shape Param #

=================================================================

input_1 (InputLayer) [(None, 120, 120, 1)] 0

_________________________________________________________________

normalization (Normalization (None, 120, 120, 1) 3

_________________________________________________________________

random_flip (RandomFlip) (None, 120, 120, 1) 0

_________________________________________________________________

random_contrast (RandomContr (None, 120, 120, 1) 0

_________________________________________________________________

conv2d (Conv2D) (None, 118, 118, 32) 320

_________________________________________________________________

conv2d_1 (Conv2D) (None, 116, 116, 64) 18496

_________________________________________________________________

dropout (Dropout) (None, 116, 116, 64) 0

_________________________________________________________________

conv2d_2 (Conv2D) (None, 114, 114, 32) 18464

_________________________________________________________________

conv2d_3 (Conv2D) (None, 112, 112, 32) 9248

_________________________________________________________________

dropout_1 (Dropout) (None, 112, 112, 32) 0

_________________________________________________________________

flatten (Flatten) (None, 401408) 0

_________________________________________________________________

dropout_2 (Dropout) (None, 401408) 0

_________________________________________________________________

dense (Dense) (None, 1) 401409

_________________________________________________________________

classification_head_1 (Activ (None, 1) 0

=================================================================

Total params: 447,940

Trainable params: 447,937

Non-trainable params: 3

_________________________________________________________________

REGRESSION OF GY USING MLP

Replication 1

_________________________________________________________________

Layer (type) Output Shape Param #

=================================================================

input_1 (InputLayer) [(None, 904)] 0

_________________________________________________________________

multi_category_encoding (Mul (None, 904) 0

_________________________________________________________________

dense (Dense) (None, 32) 28960

_________________________________________________________________

re_lu (ReLU) (None, 32) 0

_________________________________________________________________

dropout (Dropout) (None, 32) 0

_________________________________________________________________

dense_1 (Dense) (None, 32) 1056

_________________________________________________________________

re_lu_1 (ReLU) (None, 32) 0

_________________________________________________________________

dropout_1 (Dropout) (None, 32) 0

_________________________________________________________________

dropout_2 (Dropout) (None, 32) 0

_________________________________________________________________

regression_head_1 (Dense) (None, 1) 33

=================================================================

Total params: 30,049

Trainable params: 30,049

Non-trainable params: 0

_________________________________________________________________

Replication 2

_________________________________________________________________

Layer (type) Output Shape Param #

=================================================================

input_1 (InputLayer) [(None, 904)] 0

_________________________________________________________________

multi_category_encoding (Mul (None, 904) 0

_________________________________________________________________

normalization (Normalization (None, 904) 1809

_________________________________________________________________

dense (Dense) (None, 64) 57920

_________________________________________________________________

re_lu (ReLU) (None, 64) 0

_________________________________________________________________

dropout (Dropout) (None, 64) 0

_________________________________________________________________

regression_head_1 (Dense) (None, 1) 65

=================================================================

Total params: 59,794

Trainable params: 57,985

Non-trainable params: 1,809

_________________________________________________________________

Replication 3

_________________________________________________________________

Layer (type) Output Shape Param #

=================================================================

input_1 (InputLayer) [(None, 904)] 0

_________________________________________________________________

multi_category_encoding (Mul (None, 904) 0

_________________________________________________________________

dense (Dense) (None, 1024) 926720

_________________________________________________________________

re_lu (ReLU) (None, 1024) 0

_________________________________________________________________

dropout (Dropout) (None, 1024) 0

_________________________________________________________________

dense_1 (Dense) (None, 32) 32800

_________________________________________________________________

re_lu_1 (ReLU) (None, 32) 0

_________________________________________________________________

dropout_1 (Dropout) (None, 32) 0

_________________________________________________________________

dense_2 (Dense) (None, 128) 4224

_________________________________________________________________

re_lu_2 (ReLU) (None, 128) 0

_________________________________________________________________

dropout_2 (Dropout) (None, 128) 0

_________________________________________________________________

regression_head_1 (Dense) (None, 1) 129

=================================================================

Total params: 963,873

Trainable params: 963,873

Non-trainable params: 0

_________________________________________________________________

Replication 4

_________________________________________________________________

Layer (type) Output Shape Param #

=================================================================

input_1 (InputLayer) [(None, 904)] 0

_________________________________________________________________

multi_category_encoding (Mul (None, 904) 0

_________________________________________________________________

dense (Dense) (None, 256) 231680

_________________________________________________________________

batch_normalization (BatchNo (None, 256) 1024

_________________________________________________________________

re_lu (ReLU) (None, 256) 0

_________________________________________________________________

dropout (Dropout) (None, 256) 0

_________________________________________________________________

regression_head_1 (Dense) (None, 1) 257

=================================================================

Total params: 232,961

Trainable params: 232,449

Non-trainable params: 512

_________________________________________________________________

Replication 5

_________________________________________________________________

Layer (type) Output Shape Param #

=================================================================

input_1 (InputLayer) [(None, 904)] 0

_________________________________________________________________

multi_category_encoding (Mul (None, 904) 0

_________________________________________________________________

dense (Dense) (None, 32) 28960

_________________________________________________________________

re_lu (ReLU) (None, 32) 0

_________________________________________________________________

dense_1 (Dense) (None, 32) 1056

_________________________________________________________________

re_lu_1 (ReLU) (None, 32) 0

_________________________________________________________________

dense_2 (Dense) (None, 32) 1056

_________________________________________________________________

re_lu_2 (ReLU) (None, 32) 0

_________________________________________________________________

regression_head_1 (Dense) (None, 1) 33

=================================================================

Total params: 31,105

Trainable params: 31,105

Non-trainable params: 0

_________________________________________________________________

REGRESSION OF PH USING MLP

Replication 1

_________________________________________________________________

Layer (type) Output Shape Param #

=================================================================

input_1 (InputLayer) [(None, 904)] 0

_________________________________________________________________

multi_category_encoding (Mul (None, 904) 0

_________________________________________________________________

normalization (Normalization (None, 904) 1809

_________________________________________________________________

dense (Dense) (None, 1024) 926720

_________________________________________________________________

batch_normalization (BatchNo (None, 1024) 4096

_________________________________________________________________

re_lu (ReLU) (None, 1024) 0

_________________________________________________________________

dropout (Dropout) (None, 1024) 0

_________________________________________________________________

dropout_1 (Dropout) (None, 1024) 0

_________________________________________________________________

regression_head_1 (Dense) (None, 1) 1025

=================================================================

Total params: 933,650

Trainable params: 929,793

Non-trainable params: 3,857

_________________________________________________________________

Replication 2

_________________________________________________________________

Layer (type) Output Shape Param #

=================================================================

input_1 (InputLayer) [(None, 904)] 0

_________________________________________________________________

multi_category_encoding (Mul (None, 904) 0

_________________________________________________________________

normalization (Normalization (None, 904) 1809

_________________________________________________________________

dense (Dense) (None, 64) 57920

_________________________________________________________________

batch_normalization (BatchNo (None, 64) 256

_________________________________________________________________

re_lu (ReLU) (None, 64) 0

_________________________________________________________________

dropout (Dropout) (None, 64) 0

_________________________________________________________________

regression_head_1 (Dense) (None, 1) 65

=================================================================

Total params: 60,050

Trainable params: 58,113

Non-trainable params: 1,937

_________________________________________________________________

Replication 3

_________________________________________________________________

Layer (type) Output Shape Param #

=================================================================

input_1 (InputLayer) [(None, 904)] 0

_________________________________________________________________

multi_category_encoding (Mul (None, 904) 0

_________________________________________________________________

dense (Dense) (None, 32) 28960

_________________________________________________________________

re_lu (ReLU) (None, 32) 0

_________________________________________________________________

dense_1 (Dense) (None, 32) 1056

_________________________________________________________________

re_lu_1 (ReLU) (None, 32) 0

_________________________________________________________________

dropout (Dropout) (None, 32) 0

_________________________________________________________________

regression_head_1 (Dense) (None, 1) 33

=================================================================

Total params: 30,049

Trainable params: 30,049

Non-trainable params: 0

_________________________________________________________________

Replication 4

_________________________________________________________________

Layer (type) Output Shape Param #

=================================================================

input_1 (InputLayer) [(None, 904)] 0

_________________________________________________________________

multi_category_encoding (Mul (None, 904) 0

_________________________________________________________________

dense (Dense) (None, 32) 28960

_________________________________________________________________

re_lu (ReLU) (None, 32) 0

_________________________________________________________________

dense_1 (Dense) (None, 256) 8448

_________________________________________________________________

re_lu_1 (ReLU) (None, 256) 0

_________________________________________________________________

dropout (Dropout) (None, 256) 0

_________________________________________________________________

regression_head_1 (Dense) (None, 1) 257

=================================================================

Total params: 37,665

Trainable params: 37,665

Non-trainable params: 0

_________________________________________________________________

Replication 5

_________________________________________________________________

Layer (type) Output Shape Param #

=================================================================

input_1 (InputLayer) [(None, 904)] 0

_________________________________________________________________

multi_category_encoding (Mul (None, 904) 0

_________________________________________________________________

dense (Dense) (None, 32) 28960

_________________________________________________________________

re_lu (ReLU) (None, 32) 0

_________________________________________________________________

dropout (Dropout) (None, 32) 0

_________________________________________________________________

regression_head_1 (Dense) (None, 1) 33

=================================================================

Total params: 28,993

Trainable params: 28,993

Non-trainable params: 0

_________________________________________________________________

REGRESSION OF GY USING CNN

Replication 1

_________________________________________________________________

Layer (type) Output Shape Param #

=================================================================

input_1 (InputLayer) [(None, 120, 120, 1)] 0

_________________________________________________________________

normalization (Normalization (None, 120, 120, 1) 3

_________________________________________________________________

random_flip (RandomFlip) (None, 120, 120, 1) 0

_________________________________________________________________

conv2d (Conv2D) (None, 118, 118, 256) 2560

_________________________________________________________________

conv2d_1 (Conv2D) (None, 116, 116, 32) 73760

_________________________________________________________________

max_pooling2d (MaxPooling2D) (None, 58, 58, 32) 0

_________________________________________________________________

dropout (Dropout) (None, 58, 58, 32) 0

_________________________________________________________________

dropout_1 (Dropout) (None, 58, 58, 32) 0

_________________________________________________________________

flatten (Flatten) (None, 107648) 0

_________________________________________________________________

regression_head_1 (Dense) (None, 1) 107649

=================================================================

Total params: 183,972

Trainable params: 183,969

Non-trainable params: 3

_________________________________________________________________

Replication 2

_________________________________________________________________

Layer (type) Output Shape Param #

=================================================================

input_1 (InputLayer) [(None, 120, 120, 1)] 0

_________________________________________________________________

normalization (Normalization (None, 120, 120, 1) 3

_________________________________________________________________

random_flip (RandomFlip) (None, 120, 120, 1) 0

_________________________________________________________________

conv2d (Conv2D) (None, 118, 118, 16) 160

_________________________________________________________________

conv2d_1 (Conv2D) (None, 116, 116, 32) 4640

_________________________________________________________________

max_pooling2d (MaxPooling2D) (None, 58, 58, 32) 0

_________________________________________________________________

dropout (Dropout) (None, 58, 58, 32) 0

_________________________________________________________________

flatten (Flatten) (None, 107648) 0

_________________________________________________________________

regression_head_1 (Dense) (None, 1) 107649

=================================================================

Total params: 112,452

Trainable params: 112,449

Non-trainable params: 3

_________________________________________________________________

Replication 3

_________________________________________________________________

Layer (type) Output Shape Param #

=================================================================

input_1 (InputLayer) [(None, 120, 120, 1)] 0

_________________________________________________________________

conv2d (Conv2D) (None, 118, 118, 32) 320

_________________________________________________________________

conv2d_1 (Conv2D) (None, 116, 116, 256) 73984

_________________________________________________________________

conv2d_2 (Conv2D) (None, 114, 114, 32) 73760

_________________________________________________________________

conv2d_3 (Conv2D) (None, 112, 112, 32) 9248

_________________________________________________________________

flatten (Flatten) (None, 401408) 0

_________________________________________________________________

regression_head_1 (Dense) (None, 1) 401409

=================================================================

Total params: 558,721

Trainable params: 558,721

Non-trainable params: 0

_________________________________________________________________

Replication 4

_________________________________________________________________

Layer (type) Output Shape Param #

=================================================================

input_1 (InputLayer) [(None, 120, 120, 1)] 0

_________________________________________________________________

conv2d (Conv2D) (None, 118, 118, 32) 320

_________________________________________________________________

conv2d_1 (Conv2D) (None, 116, 116, 128) 36992

_________________________________________________________________

max_pooling2d (MaxPooling2D) (None, 58, 58, 128) 0

_________________________________________________________________

dropout (Dropout) (None, 58, 58, 128) 0

_________________________________________________________________

flatten (Flatten) (None, 430592) 0

_________________________________________________________________

regression_head_1 (Dense) (None, 1) 430593

=================================================================

Total params: 467,905

Trainable params: 467,905

Non-trainable params: 0

_________________________________________________________________

Replication 5

_________________________________________________________________

Layer (type) Output Shape Param #

=================================================================

input_1 (InputLayer) [(None, 120, 120, 1)] 0

_________________________________________________________________

conv2d (Conv2D) (None, 118, 118, 32) 320

_________________________________________________________________

conv2d_1 (Conv2D) (None, 116, 116, 128) 36992

_________________________________________________________________

dropout (Dropout) (None, 116, 116, 128) 0

_________________________________________________________________

flatten (Flatten) (None, 1722368) 0

_________________________________________________________________

regression_head_1 (Dense) (None, 1) 1722369

=================================================================

Total params: 1,759,681

Trainable params: 1,759,681

Non-trainable params: 0

_________________________________________________________________

REGRESSION OF PH USING CNN

Replication 1

_________________________________________________________________

Layer (type) Output Shape Param #

=================================================================

input_1 (InputLayer) [(None, 120, 120, 1)] 0

_________________________________________________________________

conv2d (Conv2D) (None, 118, 118, 128) 1280

_________________________________________________________________

conv2d_1 (Conv2D) (None, 116, 116, 32) 36896

_________________________________________________________________

max_pooling2d (MaxPooling2D) (None, 58, 58, 32) 0

_________________________________________________________________

dropout (Dropout) (None, 58, 58, 32) 0

_________________________________________________________________

flatten (Flatten) (None, 107648) 0

_________________________________________________________________

regression_head_1 (Dense) (None, 1) 107649

=================================================================

Total params: 145,825

Trainable params: 145,825

Non-trainable params: 0

_________________________________________________________________

Replication 2

_________________________________________________________________

Layer (type) Output Shape Param #

=================================================================

input_1 (InputLayer) [(None, 120, 120, 1)] 0

_________________________________________________________________

normalization (Normalization (None, 120, 120, 1) 3

_________________________________________________________________

random_flip (RandomFlip) (None, 120, 120, 1) 0

_________________________________________________________________

separable_conv2d (SeparableC (None, 118, 118, 32) 73

_________________________________________________________________

separable_conv2d_1 (Separabl (None, 116, 116, 32) 1344

_________________________________________________________________

separable_conv2d_2 (Separabl (None, 114, 114, 32) 1344

_________________________________________________________________

separable_conv2d_3 (Separabl (None, 112, 112, 32) 1344

_________________________________________________________________

flatten (Flatten) (None, 401408) 0

_________________________________________________________________

regression_head_1 (Dense) (None, 1) 401409

=================================================================

Total params: 405,517

Trainable params: 405,514

Non-trainable params: 3

_________________________________________________________________

Replication 3

_________________________________________________________________

Layer (type) Output Shape Param #

=================================================================

input_1 (InputLayer) [(None, 120, 120, 1)] 0

_________________________________________________________________

conv2d (Conv2D) (None, 118, 118, 32) 320

_________________________________________________________________

conv2d_1 (Conv2D) (None, 116, 116, 32) 9248

_________________________________________________________________

max_pooling2d (MaxPooling2D) (None, 58, 58, 32) 0

_________________________________________________________________

conv2d_2 (Conv2D) (None, 56, 56, 32) 9248

_________________________________________________________________

conv2d_3 (Conv2D) (None, 54, 54, 256) 73984

_________________________________________________________________

max_pooling2d_1 (MaxPooling2 (None, 27, 27, 256) 0

_________________________________________________________________

conv2d_4 (Conv2D) (None, 25, 25, 32) 73760

_________________________________________________________________

conv2d_5 (Conv2D) (None, 23, 23, 32) 9248

_________________________________________________________________

max_pooling2d_2 (MaxPooling2 (None, 11, 11, 32) 0

_________________________________________________________________

flatten (Flatten) (None, 3872) 0

_________________________________________________________________

regression_head_1 (Dense) (None, 1) 3873

=================================================================

Total params: 179,681

Trainable params: 179,681

Non-trainable params: 0

_________________________________________________________________

Replication 4

_________________________________________________________________

Layer (type) Output Shape Param #

=================================================================

input_1 (InputLayer) [(None, 120, 120, 1)] 0

_________________________________________________________________

random_flip (RandomFlip) (None, 120, 120, 1) 0

_________________________________________________________________

conv2d (Conv2D) (None, 118, 118, 32) 320

_________________________________________________________________

conv2d_1 (Conv2D) (None, 116, 116, 256) 73984

_________________________________________________________________

max_pooling2d (MaxPooling2D) (None, 58, 58, 256) 0

_________________________________________________________________

conv2d_2 (Conv2D) (None, 56, 56, 32) 73760

_________________________________________________________________

conv2d_3 (Conv2D) (None, 54, 54, 32) 9248

_________________________________________________________________

max_pooling2d_1 (MaxPooling2 (None, 27, 27, 32) 0

_________________________________________________________________

flatten (Flatten) (None, 23328) 0

_________________________________________________________________

regression_head_1 (Dense) (None, 1) 23329

=================================================================

Total params: 180,641

Trainable params: 180,641

Non-trainable params: 0

_________________________________________________________________

Replication 5

_________________________________________________________________

Layer (type) Output Shape Param #

=================================================================

input_1 (InputLayer) [(None, 120, 120, 1)] 0

_________________________________________________________________

separable_conv2d (SeparableC (None, 118, 118, 32) 73

_________________________________________________________________

separable_conv2d_1 (Separabl (None, 116, 116, 32) 1344

_________________________________________________________________

max_pooling2d (MaxPooling2D) (None, 58, 58, 32) 0

_________________________________________________________________

dropout (Dropout) (None, 58, 58, 32) 0

_________________________________________________________________

separable_conv2d_2 (Separabl (None, 56, 56, 64) 2400

_________________________________________________________________

separable_conv2d_3 (Separabl (None, 54, 54, 512) 33856

_________________________________________________________________

max_pooling2d_1 (MaxPooling2 (None, 27, 27, 512) 0

_________________________________________________________________

dropout_1 (Dropout) (None, 27, 27, 512) 0

_________________________________________________________________

dropout_2 (Dropout) (None, 27, 27, 512) 0

_________________________________________________________________

flatten (Flatten) (None, 373248) 0

_________________________________________________________________

regression_head_1 (Dense) (None, 1) 373249

=================================================================

Total params: 410,922

Trainable params: 410,922

Non-trainable params: 0

_________________________________________________________________
