# Supplementary material for: Automated Machine Learning: A Case Study of Genomic “Image-Based” Prediction in Maize Hybrids
Source: Front Plant Sci. 2022 Mar 7;13:845524. doi: 10.3389/fpls.2022.845524 (PMC8936805; doi:10.3389/fpls.2022.845524)
Supplement: Supplementary file 3 [file Image_1.pdf]

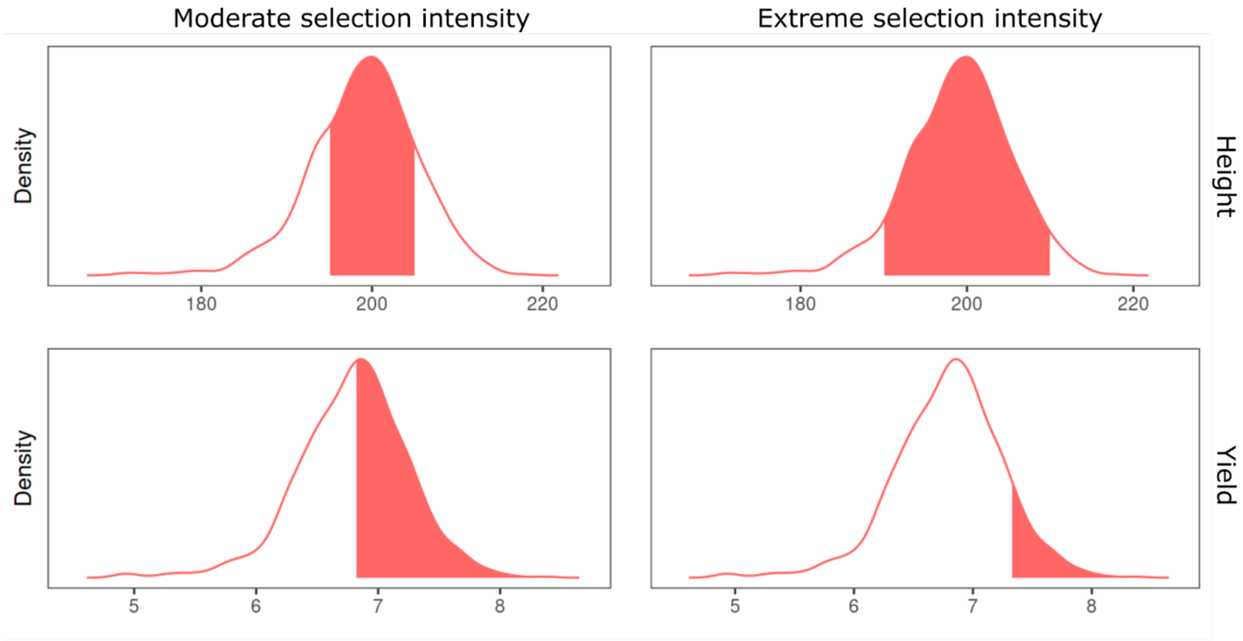

Supplemental Figure S1. Empirical distributions of genotypic values of grain yield and plant height of 904 maize hybrids. The selection was regarded using two intensities, moderate and extreme. Red areas represent the range of genotypic values considered as selected; white areas represent the non-selected genotypes. Under extreme selection intensity for GY, the 10% best were selected, while for PH, the 10% out of type were eliminated.
